# Endothelial BMAL1 decline during aging leads to bone loss by destabilizing extracellular fibrillin-1

**DOI:** 10.1172/JCI176660

**Published:** 2024-12-16

**Authors:** Ying Yin, Qingming Tang, Jingxi Yang, Shiqi Gui, Yifan Zhang, Yufeng Shen, Xin Zhou, Shaoling Yu, Guangjin Chen, Jiwei Sun, Zhenshuo Han, Luoying Zhang, Lili Chen

**Affiliations:** 1 Department of Stomatology, Union Hospital and; 2 School of Stomatology, Tongji Medical College, Huazhong University of Science and Technology, Wuhan, China.; 3 Hubei Province Key Laboratory of Oral and Maxillofacial Development and Regeneration, Wuhan, China.; 4 Key Laboratory of Molecular Biophysics of the Ministry of Education, College of Life Science and Technology, Huazhong University of Science and Technology, Wuhan, China.

**Keywords:** Aging, Bone biology, Bone disease, Cellular senescence, Extracellular matrix

## Abstract

The occurrence of aging is intricately associated with alterations in circadian rhythms that coincide with stem cell exhaustion. Nonetheless, the extent to which the circadian system governs skeletal aging remains inadequately understood. Here, we noticed that skeletal aging in male mice was accompanied by a decline in a core circadian protein, BMAL1, especially in bone marrow endothelial cells (ECs). Using male mice with endothelial KO of aryl hydrocarbon receptor nuclear translocator–like protein 1 (*Bmal1*), we ascertained that endothelial BMAL1 in bone played a crucial role in ensuring the stability of an extracellular structural component, fibrillin-1 (FBN1), through regulation of the equilibrium between the extracellular matrix (ECM) proteases thrombospondin type 1 domain–containing protein 4 (THSD4) and metalloproteinase with thrombospondin motifs 4 (ADAMTS4), which promote FBN1 assembly and breakdown, respectively. The decline of endothelial BMAL1 during aging prompted excessive breakdown of FBN1, leading to persistent activation of TGF-β/SMAD3 signaling and exhaustion of bone marrow mesenchymal stem cells. Meanwhile, the free TGF-β could promote osteoclast formation. Further analysis revealed that activation of ADAMTS4 in ECs lacking BMAL1 was stimulated by TGF-β/SMAD3 signaling through an ECM-positive feedback mechanism, whereas THSD4 was under direct transcriptional control by endothelial BMAL1. Our investigation has elucidated the etiology of bone aging in male mice by defining the role of ECs in upholding the equilibrium within the ECM, consequently coordinating osteogenic and osteoclastic activities and retarding skeletal aging.

## Abstract 2

Endothelial BMAL1 decline during aging destabilizes extracellular Fibrillin-1 by regulating THSD4 and ADAMTS4, leading to bone marrow mesenchymal stem cells exhaustion and osteoclast formation.

## Graphical abstract

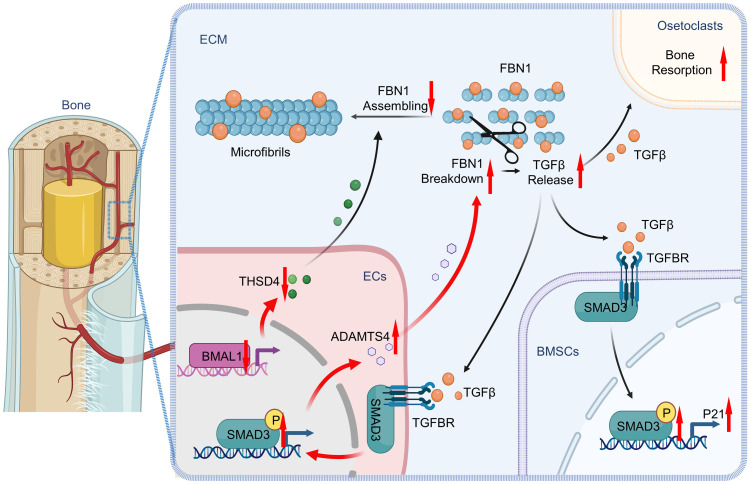

## Introduction

It has been estimated that approximately 10.3% of individuals aged 50 and older are affected by osteoporosis, whereas osteopenia is present in 54% of adults in the same age group (1). Men account for 39% of osteoporotic fracture cases (2), however, studies of skeletal aging in men have not received much attention. Previous studies have found that changes in bone aging in men include the reduction of bone marrow mesenchymal stem cells (BMSCs) and osteoblasts, the degradation of bone matrix structure, and the enhancement of osteoclast activation (3–9). Skeletal aging is inextricably associated with aging, and hormones such as testosterone and melatonin can transmit aging signals into the skeletal system, which requires the bone marrow vascular system (10–12). Bone marrow vascular endothelial cells (ECs) are closely related to osteogenic activity, and their ability to promote osteogenesis is weakened during aging (13, 14). A substantial number of studies have been conducted, but the process and fundamental mechanism underlying skeletal aging in men remain largely unknown.

Circadian rhythms are intricately linked to various aspects of behavior and physiology, including aging, for the majority of living organisms (15–18). In both aged humans and mice, there is notable dysfunction in the circadian rhythms due to increased sleep fragmentation in older individuals, reduced hormone production, etc., which manifest as a dampened and prolonged rhythmic behavior, as well as a decrease in the expression of clock genes, especially aryl hydrocarbon receptor nuclear translocator–like protein 1 (*Bmal1*, also known as *Arntl1*) (11, 19–23). As the only irreplaceable circadian clock gene, *Bmal1* is of paramount importance in thwarting the advancement of aging and preventing the occurrence of age-related diseases such as neurodegeneration, which is associated with an age-related decline in BMAL1 (24–31). The inadequacy of BMAL1 in bone tissue results in a reduction of stem cells, compromised osteogenic function, and degradation of the bone matrix (32–37). However, the how exactly BMAL1 regulates skeletal aging remains an unclear and unresolved question.

In this study, we determined that the decline of BMAL1 in bone marrow was mainly in ECs during aging and uncovered the role of endothelial BMAL1 in the progression of male skeletal aging. We demonstrated that the decline of endothelial BMAL1 affected the resilience of extracellular matrix (ECM) microfibrils, specifically fibrillin-1 (FBN1), via 2 matrix proteases: thrombospondin type 1 domain–containing protein 4 (THSD4, also known as ADAMTSL6) and metalloproteinase with thrombospondin motifs 4 (ADAMTS4). We observed that, as microfibril FBN1 broke down in the ECM, TGF-β was released from microfibrils, leading to the excessive activation of its downstream signaling pathway, as well as loss of circadian rhythms in the signaling pathways, which resulted in BMSC exhaustion and an increase in osteoclast formation. These events eventually led to accelerated bone loss in male mice during natural aging. Together, our findings suggested that the expression of BMAL1 in ECs played a pivotal role in the progression of skeletal aging in male mice, offering a promising therapeutic target for combating age-related bone loss.

## Results

### The alleviation of senile bone loss can be achieved by preventing the decline of BMAL1 induced by aging.

**Figure 1:**
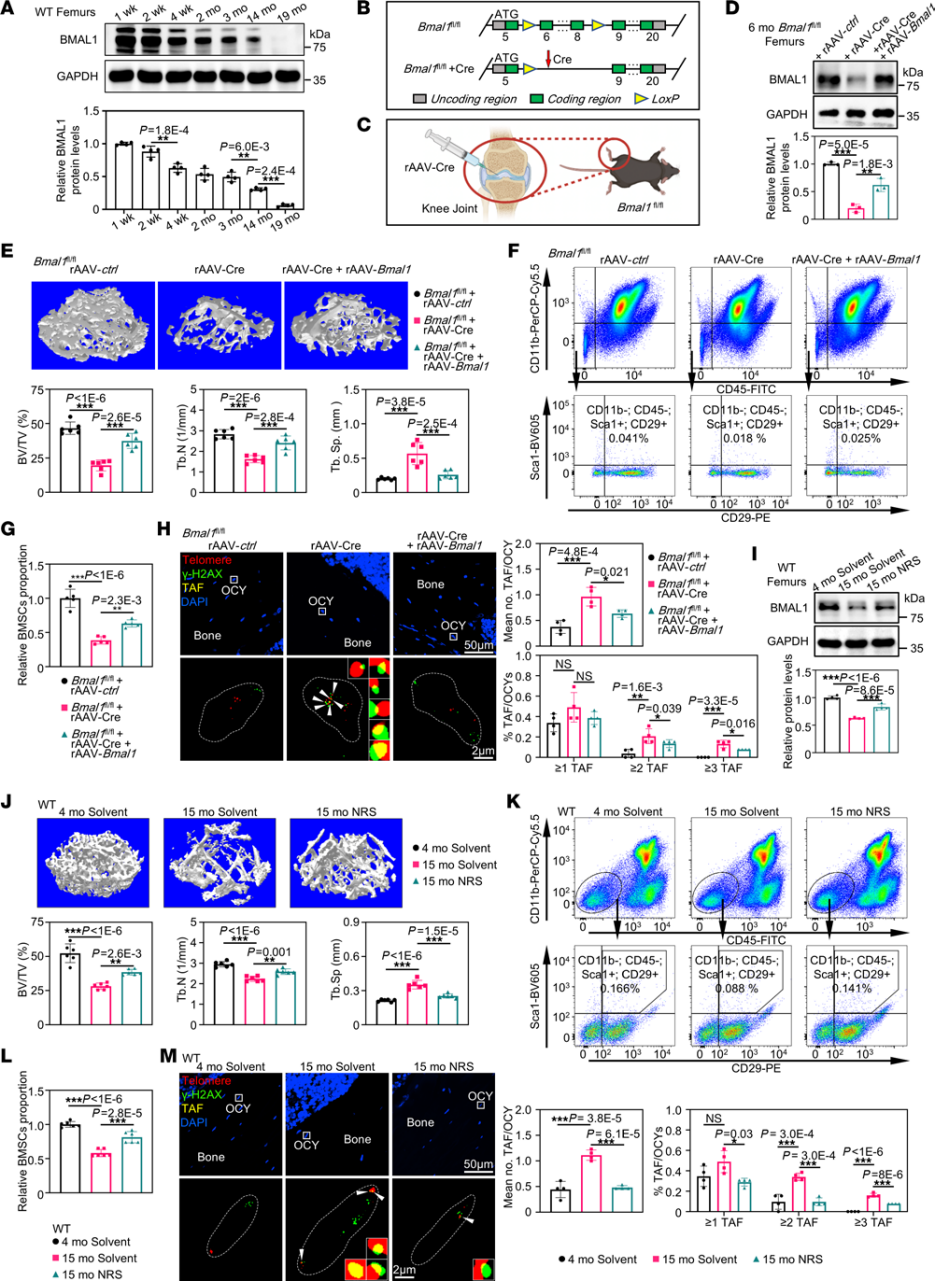
The alleviation of senile bone loss can be achieved by preventing the decline of BMAL1 induced by aging. (**A**) Western blot analysis of the levels of BMAL1 in femurs of 1-, 2-, 4-week-old and 2-, 3-, 14-, 19-month-old mice, with quantitative data (*n* = 4). (**B**) Schematic diagram of the *Bmal1*-KO (*Bmal1^fl/fl^*) mouse model. (**C**) Schematic diagram of injection of the rAAV virus into knee joints. (**D**) Western blot analysis of BMAL1 protein levels in femurs and quantitative data. rAAV-*ctrl* or rAAV-Cre was injected into mice at 3 months of age, while rAAV-Cre was injected at 5 months of age (*n* = 3). (**E**) Representative images of micro-CT reconstruction of femurs corresponding to the samples in **D**, with quantitative data shown (*n* = 6). (**F**) Representative flow cytometry plot showing CD11^–^CD45^–^Sca1^+^CD29^+^ BMSCs corresponding to the samples in **D**. (**G**) Quantitative data for the flow cytometric results in **F** (*n* = 5). (**H**) Representative images of nonsenescent and senescent osteocyte in mice according to the TAF (white arrows) assay corresponding to the samples in **D**, with quantitative data (*n* = 4). (**I**) Western blot analysis of the BMAL1 protein levels in femurs of 4- and 15-month-old mice treated with solvent or NRS (3 mg/kg/d, oral administration) for 1 month, with quantitative data shown (*n* = 4). (**J**) Representative images of micro-CT reconstruction of femurs corresponding to the samples in **I**, with quantitative data (*n* = 6). (**K**) Representative flow cytometry plot showing CD11b^–^CD45^–^Sca1^+^CD29^+^ BMSCs corresponding to the samples in **I**. (**L**) The quantitative data for **K** (*n* = 6). (**M**) Representative images of nonsenescent and senescent osteocyte in mice according to the TAF (white arrows) assay corresponding to the samples in **I**, with quantitative data on the right (*n* = 4). Scale bars: 2 μm and 50 μm. *P < 0.05, **P < 0.01, and ***P < 0.001. Data in **A**, **D**, **E**, **F**, **H**, **I**, **J**, **L**, and **M** were analyzed by 1-way ANOVA with Tukey’s multiple-comparison test.

BMAL1 is a critical factor in hindering the advancement of aging and preventing the emergence of age-related diseases (25–29, 38). To explore the potential correlation between skeletal aging and alterations in BMAL1 expression, our initial investigation involved examining BMAL1 protein levels in the femurs of mice ranging in age from 1 week to 19 months. As expected, the results showed a marked decline in BMAL1 levels in the femurs of aged mice (Figure 1A). Next, to test the effect of reduced BMAL1 levels on femurs, instead of using a systematic *Bmal1*-KO mice that may affect other tissues and organs causing an indirect effect on bone, we generated *Bmal1^fl/fl^* mice and locally injected recombinant adeno-associated virus vectors expressing Cre (rAAV-Cre) to delete femoral *Bmal1* (Figure 1B). Moreover, to avoid harming cortical bone, we injected rAAV-Cre into the knee joints for *Bmal1* deletion, a rAAV delivery method used previously (39) (Figure 1C). rAAV-Cre was injected into *Bmal1^fl/fl^* mice at the age of 3 months for *Bmal1* deletion, and for rescue experiments, rAAV expressing *Bmal1* (rAAV-*Bmal1*) was injected again into the mice at the age of 5 months to restore the expression of *Bmal1*. Subsequently, femur samples were taken from the mice at the age of 6 months to assess BMAL1 alterations. The efficacy of *Bmal1* KO and recovery was subsequently evaluated by immunofluorescence staining and Western blot analysis (Figure 1D and Supplemental Figure 1A; supplemental material available online with this article; https://doi.org/10.1172/JCI176660DS1). Bone assessment showed that *Bmal1* KO induced a marked acceleration in femoral bone loss, accompanied by diminished biomechanical strength (Figure 1E and Supplemental Figure 1B). Consistent with bone loss, *Bmal1^fl/fl^* mice that received injections of rAAV-Cre had fewer osteoblasts and more osteoclasts (Supplemental Figure 1, C and D). Furthermore, flow cytometry showed that KO of *Bmal1* resulted in a marked decrease in the number of CD11b^–^CD45^–^Sca1^+^CD29^+^ BMSCs, whereas *Bmal1* deletion showed a noticeable rise in telomere damage of femoral osteocytes (Figure 1, F–H, and Supplemental Figure 1J). In addition, the femurs of *Bmal1*-KO mice exhibited an increase in the expression of genes related to senescence-associated secretory phenotype (SASP) (Supplemental Figure 1E). However, restoring *Bmal1* in the femurs could effectively alleviate the above bone aging–related phenotypes. In summary, our results supported the notion that the decline in BMAL1 expression initiates the early manifestation of age-related changes in the skeletal system.

The data we had acquired thus far led us to speculate that increasing BMAL1 levels may counteract the effects of aging on the skeletal structure. To test this possibility, we treated aged mice at 14 months with neoruscogenin (NRS), a BMAL1 agonist, for 1 month (40), and first confirmed its effect on BMAL1 protein levels (Figure 1I). Next, we found that aged mice with NRS treatment had more osteoblasts and fewer osteoclasts, with increased bone mass and enhanced biomechanical strength in the femurs (Figure 1J and Supplemental Figure 1, F–H). Moreover, NRS markedly increased the number of BMSCs (CD11b–CD45–Sca1^+^CD29^+^), while simultaneously reducing femoral osteocyte telomere damage (Figure 1, K–M). The expression of genes related to SASP was also restored to a level comparable to that in 4-month-old mice upon NRS treatment (Supplemental Figure 1I). These results suggested that NRS could effectively reverse age-related changes in the skeletal system. Taken together, our findings provided evidence that BMAL1 played a critical role in maintaining skeletal health in aged mice.

### Premature aging of the skeletal system is caused by the loss of BMAL1 in bone marrow vascular ECs.

**Figure 2:**
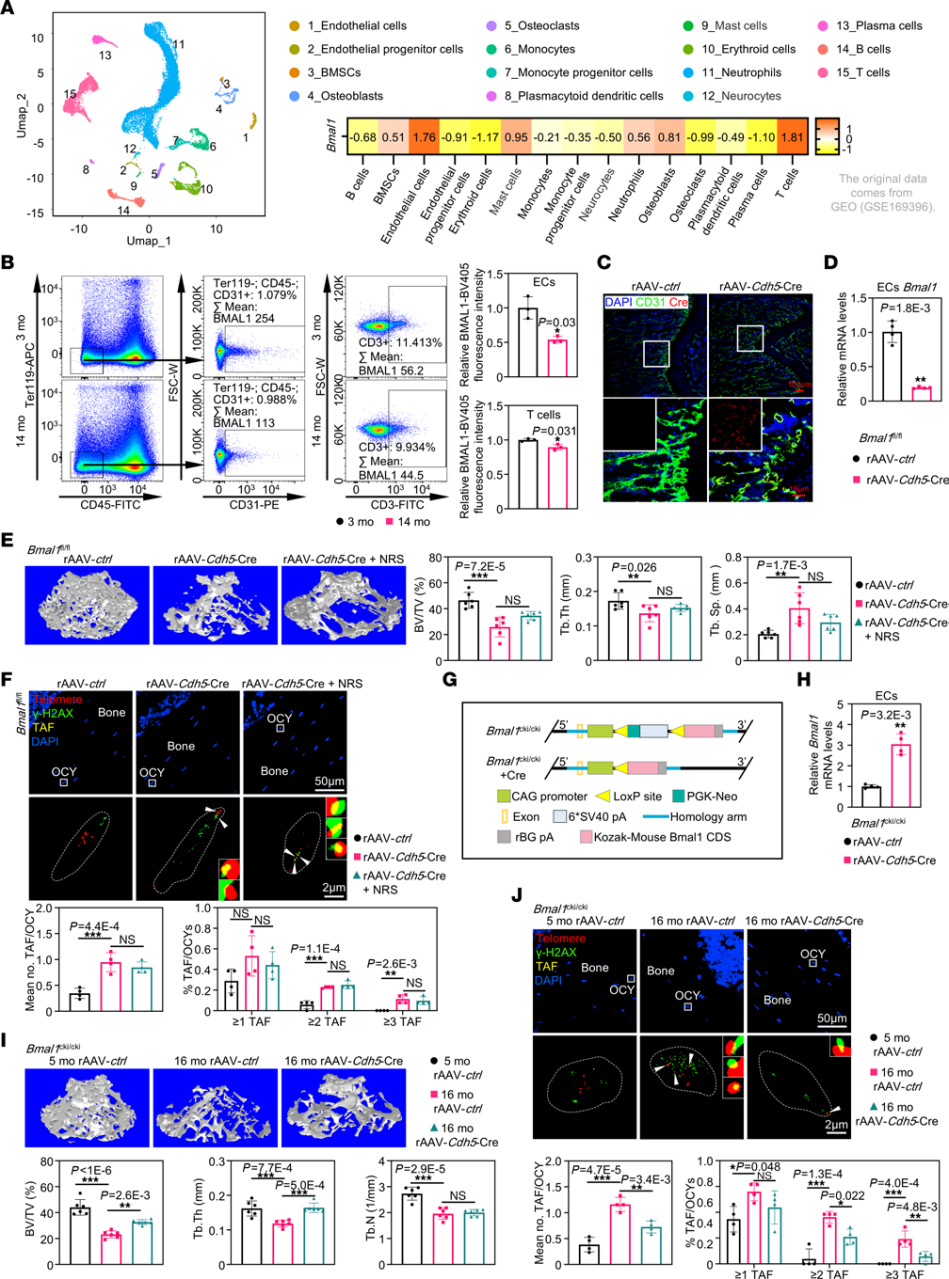
The premature aging of the skeletal system is caused by the loss of BMAL1 in bone marrow vascular ECs. (**A**) The uniform manifold approximation and projection (UMAP) plot of long bone cells from adult bone marrow (GSE169396), with a heatmap showing the mean expression of BMAL1 in various cells. (**B**) Representative flow cytometric analysis of BMAL1 levels of the Ter119^–^CD45^–^CD31^+^ vascular EC population and the CD3^+^ T cell population in bone marrow from 3- and 14-month-old mice, with quantitative data shown (*n* = 3). (**C**) Immunofluorescence images of CD31 and Cre in femurs (*n* = 3). Scale bars: 100 μm and 10 μm (enlarged insets). (**D**) qPCR data showing relative mRNA levels of *Bmal1* in ECs sorted form bone marrow ( *n* = 3). (**E**) Representative images of micro-CT reconstruction of femurs from 6-month-old *Bmal1^fl/fl^* mice, with quantitative data. Mice were injected with rAAV-*ctrl* or rAAV-*Cdh5*-Cre at 3 months of age and treated with NRS (3 mg/kg/d, per os) at 5 months of age for 1 month (*n* = 6). (**F**) Representative images of nonsenescent and senescent osteocytes in mice according to the TAF (white arrows) assay corresponding to the samples in **E**, with quantitative data (*n* = 4). Scale bars: 50 μm and 2 μm (enlarged inset). (**G**) Schematic diagram of the *Bmal1*-knockin (*Bmal1^cki/cki^*) mouse model. (**H**) qPCR quantitation data showing relative mRNA levels of *Bmal1* in ECs sorted from bone marrow (*n* = 4). (**I**) Representative images of micro-CT reconstruction of femurs from 3- or 14-month-old *Bmal1^cki/cki^* mice treated with rAAV-*ctrl* or rAAV-*Cdh5*-Cre, with quantitative data (*n* = 6). (**J**) Representative images of nonsenescent and senescent osteocytes in mice according to the TAF (white arrows) assay, corresponding to the samples in **I**, with quantitative data (*n* = 4). *P < 0.05, **P < 0.01, and ***P < 0.001. Data in **B**, **D**, and **H** were analyzed by unpaired, 2-tailed *t* test with Welch’s correction. Data in **E**, **F**, **I**, and **J** were analyzed by 1-way ANOVA with Tukey’s multiple-comparison test.

To determine the cell type responsible for BMAL1’s role in skeletal aging, we evaluated the distribution of BMAL1 expression in bones by analyzing single-cell RNA-Seq data (GSE169396). The sequencing data analysis demonstrated that BMAL1 was heavily expressed in ECs and T cells (Figure 2A). Consequently, we analyzed ECs and T cells collected from femurs in young and aged mice using flow cytometry to compare their BMAL1 expression levels. We found that the expression of BMAL1 had a more marked age-related decline in ECs compared with T cells (Figure 2B). To verify the effect of endothelial BMAL1 on skeletal aging, we generated mice with EC-specific depletion of *Bmal1* in femurs by injecting rAAV-*Cdh5*-Cre (Cre was only expressed in CDH5^+^ cells) into the knee joints of 3-month-old *Bmal1^fl/fl^* mice. We confirmed the exclusive expression of Cre in CD31^+^ cells, which marked for ECs, as well as the effectiveness of *Bmal1* deletion in sorted ECs from femoral bone marrow (Figure 2, C and D). We observed marked bone mass loss and fewer osteoblasts in the femurs of 6-month-old *Bmal1^fl/fl^* mice injected with rAAV-*Cdh5*-Cre, as well as reduced biomechanical properties (Figure 2E and Supplemental Figure 2, A and B). Moreover, the number of femoral osteoclasts was also slightly increased in mice with endothelial *Bmal1* depletion (Supplemental Figure 2C). Telomere-associated foci (TAF) staining revealed that the absence of endothelial BMAL1 facilitated the production of senescence cells within the femurs (Figure 2F). Consistent with more TAFs, we observed the upregulation of genes related to SASP in the femurs of *Bmal1^fl/fl^* mice injected with rAAV-*Cdh5*-Cre (Supplemental Figure 2D). To test whether NRS could reverse these effects, 2 months after rAAV-*Cdh5*-Cre injection, we administered NRS daily for 1 month to upregulate femoral BMAL1 expression. Nevertheless, NRS was unable to reverse premature aging in young endothelial *Bmal1*–deficient mice, due to BMAL1 deletion and thus no BMAL1 activation by NRS (Figure 2, E and F, and Supplemental Figure 2, A–D). In addition, flow cytometric analysis of EC numbers in the femurs of *Bmal1^fl/fl^* mouse injected with rAAV-*Cdh5*-Cre showed no marked difference (Supplemental Figure 2E). Furthermore, the in vivo experiments provided additional evidence that the depletion of *Bmal1* in ECs did not have any effect on vascular leakiness (Supplemental Figure 2F).

To validate the hypothesis that endothelial BMAL1 may serve as a safeguard against skeletal aging in mice, we generated 14-month-old mice with conditional knockin of *Bmal1* (*Bmal1^cki/cki^*) and induced the upregulation of activated endothelial *Bmal1* by injection of rAAV-*Cdh5*-Cre into knee joints (Figure 2, G and H). After 2 months of treatment, we observed a marked improvement in bone mass among aged mice, with enhanced biomechanical strength, more osteoblasts, and a mild decrease in osteoclasts (Figure 2I and Supplemental Figure 3, A–C). The aged mice, upon receiving rAAV-*Cdh5-Bmal1*, demonstrated a decline in the number of senescent cells and downregulation of SASP genes (Figure 2J and Supplemental Figure 3D). Collectively, these data demonstrated that endothelial BMAL1 played a crucial role in the regulation of skeletal aging and that elevating its levels could lead to a marked reduction in bone loss.

### BMAL1 in bone marrow vascular ECs regulates BMSC senescence.

**Figure 3:**
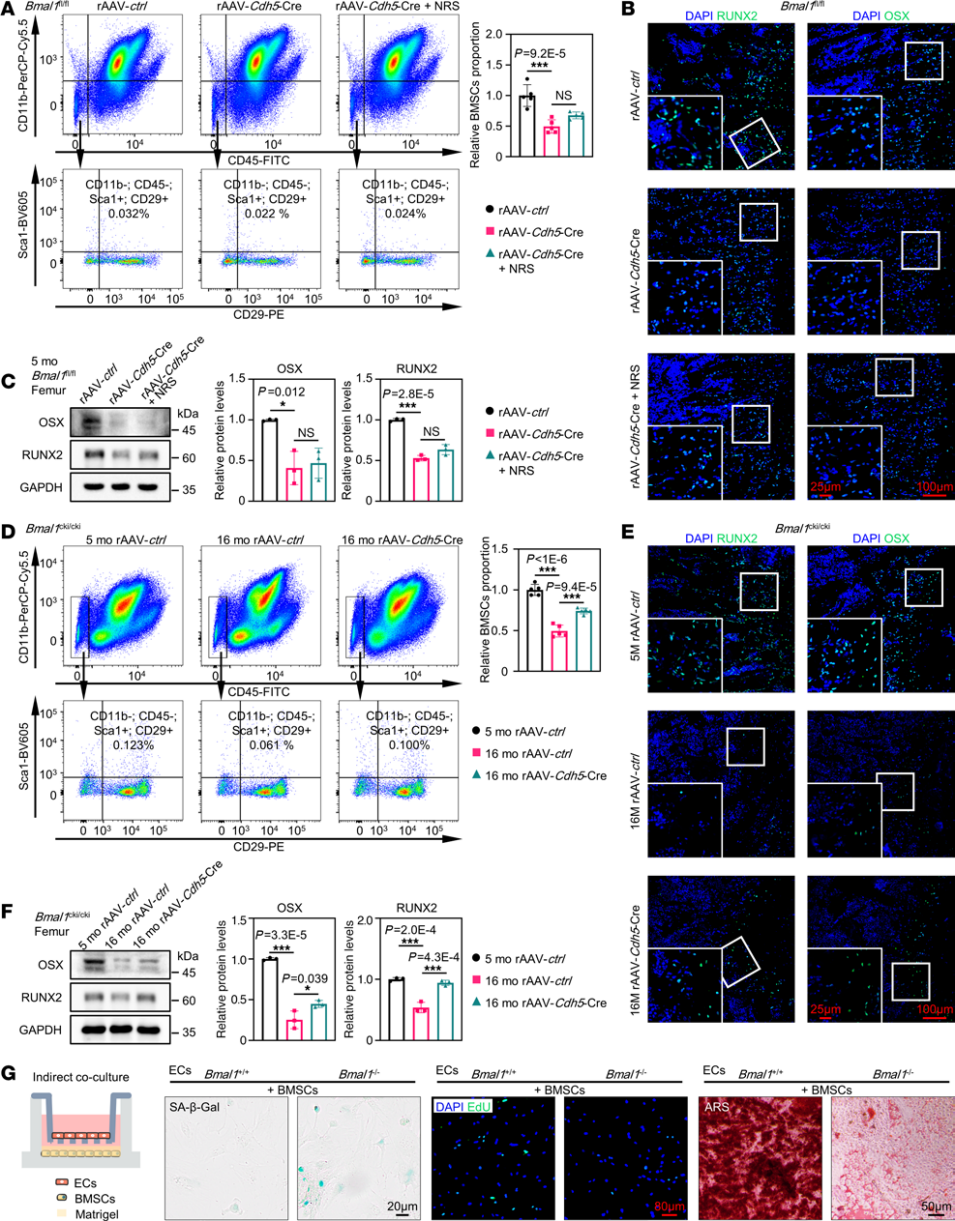
BMAL1 in bone marrow vascular ECs regulates BMSC proliferation and differentiation. (**A**) Representative flow cytometry plot showing CD11b^–^CD45^–^Sca1^+^CD29^+^ BMSCs in bone marrow corresponding to the samples in Figure 2E, with quantitative data shown (*n* = 5). (**B**) Immunofluorescence images of RUNX2 and OSX of femurs corresponding to the samples in **A** (*n* = 3). Scale bars: 100 μm and 25 μm (enlarged insets). (**C**) Western blot analysis of the levels of RUNX2 and OSX levels in femurs corresponding to the samples in **A**, with quantitative data (*n* = 3). (**D**) Representative flow cytometry plot showing CD11b^–^CD45^–^Sca1^+^CD29^+^ BMSCs in bone marrow corresponding to the samples in Figure 2I, with quantitative data (*n* = 5). (**E**) Immunofluorescence of RUNX2 and OSX in femurs corresponding to the samples in **D**. *n* = 3. (**F**) Western blot analysis of the levels of RUNX2 and OSX of femurs corresponding to the samples in **D**, with quantitative data shown (*n* = 3). Scale bars: 100 μm and 25 μm (enlarged insets). (**G**) Schematic diagram of indirect coculturing of ECs and BMSCs in Matrigel and representative SA–β-gal staining (blue), 5-ethynyl-2′-deoxyuridine (EdU) immunofluorescence, and Alizarin Red S (ARS) staining of BMSCs. ECs were sorted from the *Bmal1^fl/fl^* mice injected with rAAV-*ctrl* or rAAV-*Cdh5*-Cre (*n* = 3). Scale bars: 20 μm, 50 μm, and 80 μm. *P < 0.05 and ***P < 0.001. Data in **A**, **C**, **D**, and **F** were analyzed by 1-way ANOVA with Tukey’s multiple-comparison test.

Stem cell exhaustion can lead to a decline in the generation of new bone tissue and promote skeletal aging (6). To test whether endothelial BMAL1 loss could lead to BMSC exhaustion, we used flow cytometry to measure BMSC numbers in femurs. Consistent with our expectation, a large shrinkage in the number of BMSCs was observed in endothelial *Bmal1*–deficient mice (Figure 3A), which may have an effect on osteogenic differentiation. Indeed, immunofluorescence staining and Western blotting revealed that endothelial *Bmal1*–deficient mice had marked impairment in osteogenic function, which was characterized by a decrease in the expression of critical osteoblast differentiation markers, including Runt-related transcription factor 2 (RUNX2) and the transcription factor SP7 (OSX) (Figure 3, B and C). Conversely, through the upregulation of *Bmal1* in ECs in aged mice, we managed to increase BMSC numbers (Figure 3D). Consistent with the increase in BMSC numbers, the osteogenesis markers in aged mice were markedly upregulated (Figure 3, E and F).

Next, to investigate how a decline in endothelial BMAL1 decreases BMSC numbers, we sorted ECs and BMSCs from femurs to carry out indirect coculture experiments in vitro. The findings indicated that KO of *Bmal1* in ECs could lead to the acceleration of BMSC aging, and also impede BMSC proliferation and osteogenic differentiation via secretion of extracellular substances (Figure 3G). Moreover, colony formation assays and CCK8 assays showed that endothelial *Bmal1* deficiency could lead to decreased proliferation of BMSCs (Supplemental Figure 4, A and B). Oil red O and alizarin red staining showed that loss of endothelial BMAL1 could lead to weakening of the lipogenic differentiation of BMSCs (Supplemental Figure 4C and Figure 3G). Using flow cytometry, we found that endothelial BMAL1 loss resulted in more BMSCs stuck in the G_0_/G_1_ phase, increased ROS levels, and more apoptotic cells than in the control group (Supplemental Figure 4, D–F). In summary, the depletion of *Bmal1* in ECs, causing bone mass decline, may be primarily due to stem cell exhaustion and reduced osteogenesis.

### The regulation of BMSC senescence by endothelial BMAL1 is mediated by the matrix proteases THSD4 and ADAMTS4.

**Figure 4:**
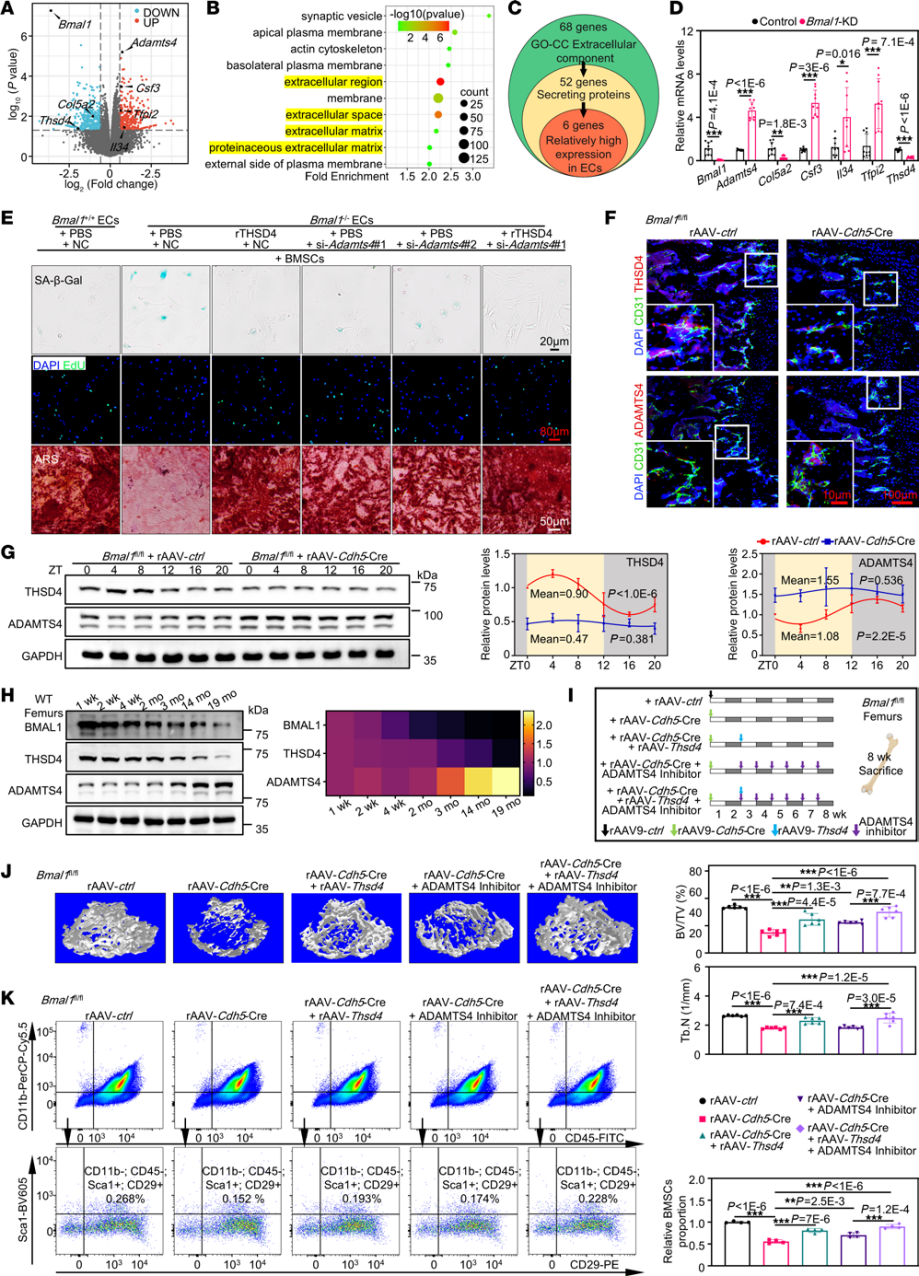
Regulation of BMSCs senescence by endothelial BMAL1 is mediated by the matrix proteases THSD4 and ADAMTS4. (**A**) Volcano plot showing the DEGs in ECs sorted from *Bmal1*-KD and control mice (*n* = 3). (**B**) Gene Ontology (GO) cellular component (CC) analysis of DEGs between *Bmal1*-KD and control ECs. (**C**) Logic diagram of screening candidate genes of secretory proteins that have relatively higher expression in ECs than other cells in bone tissue, according to the Human Protein Atlas. Genes are listed in Supplemental Table 5. (**D**) Confirmation of the candidate genes by qPCR (*n* = 3). (**E**) Representative SA–β-gal staining (blue), EdU immunofluorescence, and ARS staining of BMSCs that were indirectly cocultured with EC (*n* = 3). Scale bars: 20 μm, 50 μm, and 80 μm. (**F**) Representative images of FISH THSD4 and immunofluorescence staining for CD31 or ADAMTS4 (*n* = 3). Scale bars: 100 μm and 10 μm (insets). (**G**) Western blot analysis of the relative levels of THSD4 and ADAMTS4 in femurs over a 1-day period with quantitative data shown (*n* = 3). (**H**) Western blot analysis of BMAL1, THSD4, and ADAMTS4 protein levels at the indicated time points in femurs, with the corresponding heatmap shown (*n* = 3). (**I**) Schematic diagram of animal experiments with *Bmal1^fl/fl^* mice injected with rAAV-*ctrl*, rAAV-*Cdh5*-Cre, and rAAV-*Thsd4*, or the ADAMTS4 inhibitor. (**J**) Representative images of micro-CT reconstruction of femurs corresponding to the samples in **I**, with quantitative data shown (*n* = 6). BV/TV, bone volume over total volume; Tb.N, trabecular number. (**K**) Representative flow cytometry plot showing CD11b^–^CD45^–^Sca1^+^CD29^+^ BMSCs in bone marrow corresponding to the samples in **I**, with quantitative data shown. Shadow, night; yellow, day; ZT0, 8 am, light turned on. *n* = 4. *P < 0.05, **P < 0.01, and ***P < 0.001. Data in **D** were analyzed by unpaired, 2-tailed *t* test with Welch’s correction; data in **G** were analyzed using DiscoRhythm (cosinor analysis); and data in **J** and **K** were analyzed by 1-way ANOVA with Tukey’s multiple-comparison test.

Extracellular vesicles (EVs) are an important pathway for indirect communication between cells, and the literature has reported that ECs can secrete EVs to regulate BMSCs (41–44). We intervened in the coculture experiments using the small-molecule compounds GW4869 and imipramine, which could inhibit the formation and secretion of EVs, and found that EVs did not participate in the regulation of BMSCs by endothelial BMAL1 (Supplemental Figure 5). Then, we conducted genome-wide RNA-Seq analysis to obtain the transcriptional profile of ECs with *Bmal1* knockdown (KD). These cells were isolated directly from mouse femurs with endothelial *Bmal1* KD by injecting rAAV-*Tek*-*shBmal1* (*Tek*, an EC-specific promoter, including 2 *shBmal1*). According to the data, 684 genes exhibited differential expression levels between ECs with *Bmal1* KD and those without any intervention (Figure 4A). Subsequently, our investigation focused on identifying differentially expressed genes (DEGs) that encode secretory proteins and have been confirmed to exhibit elevated expression levels in ECs, relative to other cells present in bone, as per the Human Protein Atlas. It is important to note that we excluded genes associated with EVs from our analysis (Figure 4, B and C). Following a thorough evaluation, 6 genes were considered as suitable candidates (Figure 4D). Next, we performed a series of in vitro assessments to identify proteins responsible for the regulation of *Bmal1* in ECs in terms of senescence, proliferation, and differentiation of BMSCs. We also identified 2 matrix proteases, namely THSD4 (also known as ADAMTSL6) and ADAMTS4, as potential proteins that effectively mediated the regulation of endothelial *Bmal1* for BMSC senescence, proliferation, and osteogenic differentiation (Figure 4E and Supplemental Figure 6, A–D).

In accordance with the in vitro findings, *Bmal1^fl/fl^* mice injected with rAAV-*Cdh5*-Cre exhibited a decrease in THSD4 expression and an increase in ADAMTS4 expression when compared with expression in the controls (Figure 4F). In vivo, THSD4 and ADAMTS4 exhibited circadian oscillations, which were absent in endothelial *Bmal1*–deficient mice (Figure 4G), suggesting that THSD4 and ADAMTS4 were potentially under the control of the circadian rhythm, most likely by endothelial BMAL1. In addition, our research revealed that the BMAL1 decline during aging was accompanied by a reduction in THSD4 expression and an increase in ADAMTS4 expression (Figure 4H). To further verify the role of these 2 proteins in vivo, we administered rAAV-*Thsd4* and an ADAMTS4 inhibitor to endothelial *Bmal1*–deficient mice, thereby reinstating THSD4 expression and/or inhibiting ADAMTS4 activity (Figure 4I). The endothelial *Bmal1*–deficient mice with THSD4 restoration or ADAMTS4 inhibition showed a resurgence of bone mass and a rise in the number of stem cells, as well as more femoral bone mass and increased femoral BMSC numbers after simultaneous overexpression of THSD4 and inhibition of ADAMTS4 function (Figure 4, J and K). In addition, we also induced overexpression of THSD4 in WT aged mice and ADAMTS4 in young mice to verify their effects, respectively (Supplemental Figure 7, A, B, E, and F). After overexpression of THSD4 in aged mice, we found that both bone mass and BMSC numbers were restored (Supplemental Figure 7, C and D). Upregulation of ADAMTS4 in young mice could markedly reduce bone mass and BMSC numbers (Supplemental Figure 7, G and H).

Considering the recognized role of BMAL1 (45) as a transcription factor, we first explored the direct regulatory role of BMAL1 in *Thsd4*. We analyzed BMAL1 binding in ECs by ChIP-Seq and found 663 peaks in promoter regions and 3 peaks in the promoter of *Thsd4* (Supplemental Figure 8, A and B). E-box elements (CACGTG) were detected in the 3 peak regions of *Thsd4* (Supplemental Figure 8C). Through ChIP-qPCR and a luciferase reporter assay using *Thsd4* promoter mutants, we determined that BMAL1 was capable of binding to the promoter of *Thsd4* and activating its transcription in ECs (Supplemental Figure 8, C and D). Together, these findings suggested that the effect of endothelial BMAL1 on the BMSCs was achieved through direct activation of *Thsd4* expression and a potential indirect mechanism that inhibited ADAMTS4 expression.

### An imbalance between THSD4 and ADAMTS4 results in degradation of the extracellular structural component FBN1.

**Figure 5:**
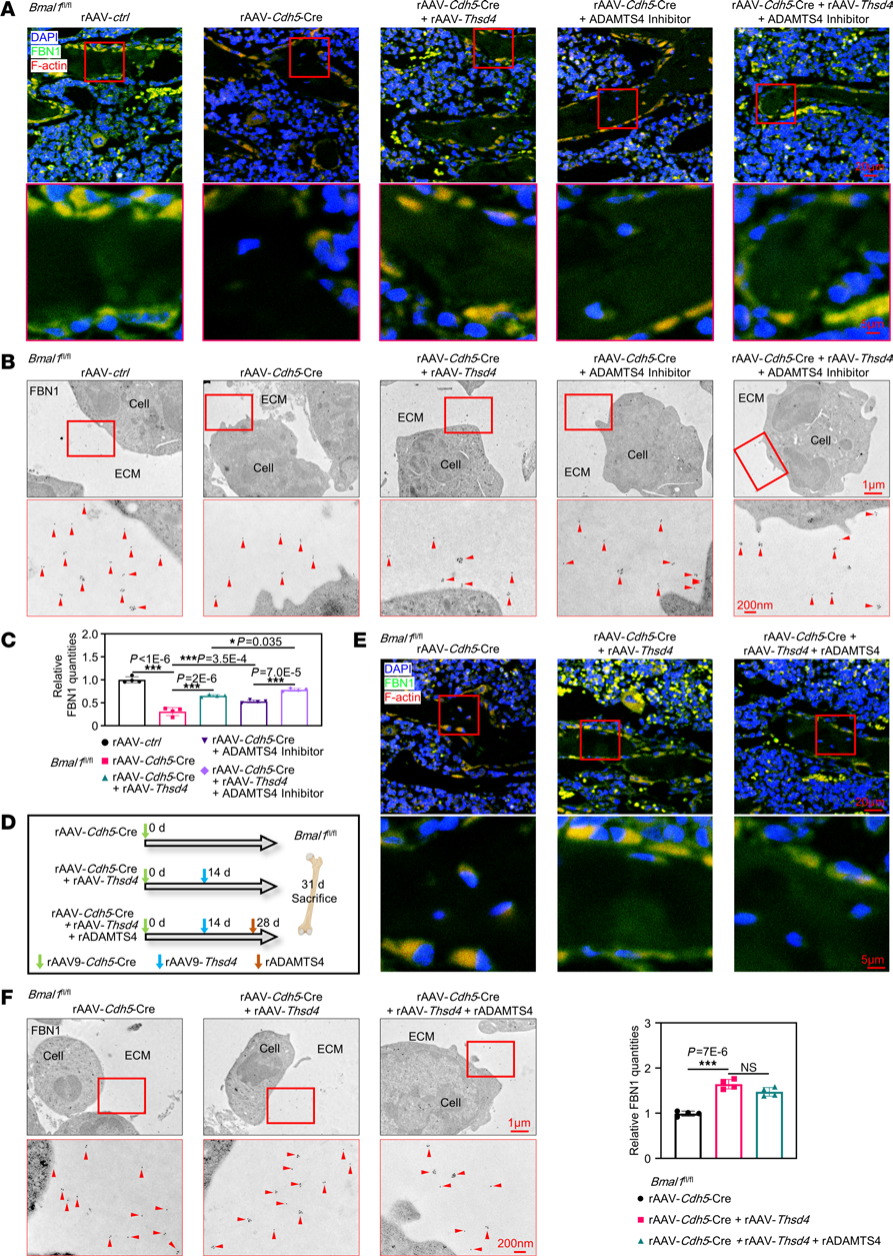
An imbalance in THSD4/ADAMTS4 signaling results in the degradation of the extracellular structural component FBN1. (**A**) Immunofluorescence of FBN1 and F-actin in femurs corresponding to the samples in Figure 4I (*n* = 3). Scale bars: 20 μm and 5 μm (enlarged insets). (**B**) Immunogold labeling of FBN1 in femurs corresponding to the samples in Figure 4I. Red arrowheads indicate FBN1 fibers. Scale bars: 1 μm and 200 nm (enlarged insets). (**C**) Quantitative data for the images in **B** (*n* = 4). (**D**) Schematic diagram of animal experiments in *Bmal1* *^fl/fl^* mice injected with rAAV-*Cdh5*-Cre, and rAAV-*Thsd4* or rADAMTS4. (**E**) Immunofluorescence images of FBN1 and F-actin in femurs corresponding to the samples in **D** (*n* = 3). Scale bars: 20 μm and 5 μm (enlarged insets). (**F**) Immunogold labeling of FBN1 in femurs corresponding to the samples in **D**, with quantitative data shown (*n* = 4). Red arrowheads indicate FBN1 fibers. *P < 0.05 and ***P < 0.001. Data in **C** and **F** were analyzed by 1-way ANOVA with Tukey’s multiple-comparison test.

FBN1, an important macromolecular component in the ECM, is widely distributed in bone tissue and has important physiological functions as a supporting structure that maintains tissue integrity and as a warehouse that stores a large amount of cytokines, such as TGF-β (46, 47). The protein THSD4 can interact with FBN1 in a direct manner by promoting the assembly of extracellular FBN1 monomers into polymers (48). Conversely, ADAMTS4 has a wide range of hydrolytic substrates, and the cleavage sites for these substrates have been identified (49, 50). In light of these clues, we propose that these 2 proteases might operate jointly on the same substrate, FBN1. To test this hypothesis, we identified a marked number of potential ADAMTS4 cleavage sites present in FBN1 (Supplemental Figure 9A). Consistent with our hypothesis, after incubating recombinant proteins of ADAMTS4 and FBN1 for a specific period, it was evident that rFBN1 underwent cleavage by rADAMTS4 (Supplemental Figure 9B). Similarly, the introduction of rADAMTS4 into the femurs of mice resulted in a marked depletion of extracellular FBN1 (Supplemental Figure 9C). Moreover, through immunofluorescence staining and immunosorbent electron microscope (ISEM) analysis, we discovered that the removal of *Bmal1* from ECs obstructed the aggregation of extracellular FBN1 monomers and promoted their degradation (Figure 5, A–C). We also examined the levels of additional matrix proteases, specifically matrix metalloproteinase 2 (MMP2) and MMP9, which were documented to possess the capability of cleaving FBN1 fibers (51, 52), and found that there was no noteworthy alteration in the femurs of endothelial *Bmal1*–deficient mice (Supplemental Figure 9, D–F). Notably, the expression of FBN1 in BMSCs was not influenced by endothelial BMAL1 depletion (Supplemental Figure 9G). Together, these data suggested a notable connection between bone matrix FBN1 and the proteases THSD4 and ADAMTS4.

To gain a better understanding of the cooperative roles of THSD4 and ADAMTS4 in bone matrix FBN1, we administered rAAV-*Thsd4* and/or an inhibitor of ADAMTS4 via injecting into knee joints to conduct in vivo experiments (Figure 4I). Eight weeks after the injection, immunofluorescence staining revealed that *Thsd4* upregulation had a markedly inhibitory effect on the decrease in extracellular FBN1, and we confirmed the formation of FBN1 microfibrils through ISEM analysis (Figure 5, A–C). Following treatment with an ADAMTS4 inhibitor, femurs exhibited a marked elevation in FBN1 within the bone matrix (Figure 5, A–C). To examine the combinatory effect of THSD4 and ADAMTS4 on extracellular FBN1, we injected rAAV-*Thsd4* and recombinant ADAMTS4 into the knees of endothelial *Bmal1*–deficient mice (Figure 5D). The addition of rADAMTS4 did not notably promote FBN1 hydrolysis in mice with decreased endothelial BMAL1 that were injected with rAAV-Thsd4 (Figure 5, E and F). This suggests that once FBN1 monomers are assembled into microfibrils, they may acquire the ability to resist ADAMTS4 pyrolysis. Together, these data demonstrated that the absence of endothelial BMAL1 may result in an imbalance of THSD4/ADAMTS4 signaling, subsequently leading to the degradation of extracellular FBN1.

### Degradation of FBN1 triggers persistent activation of TGF-β signaling, leading to BMSC exhaustion.

**Figure 6:**
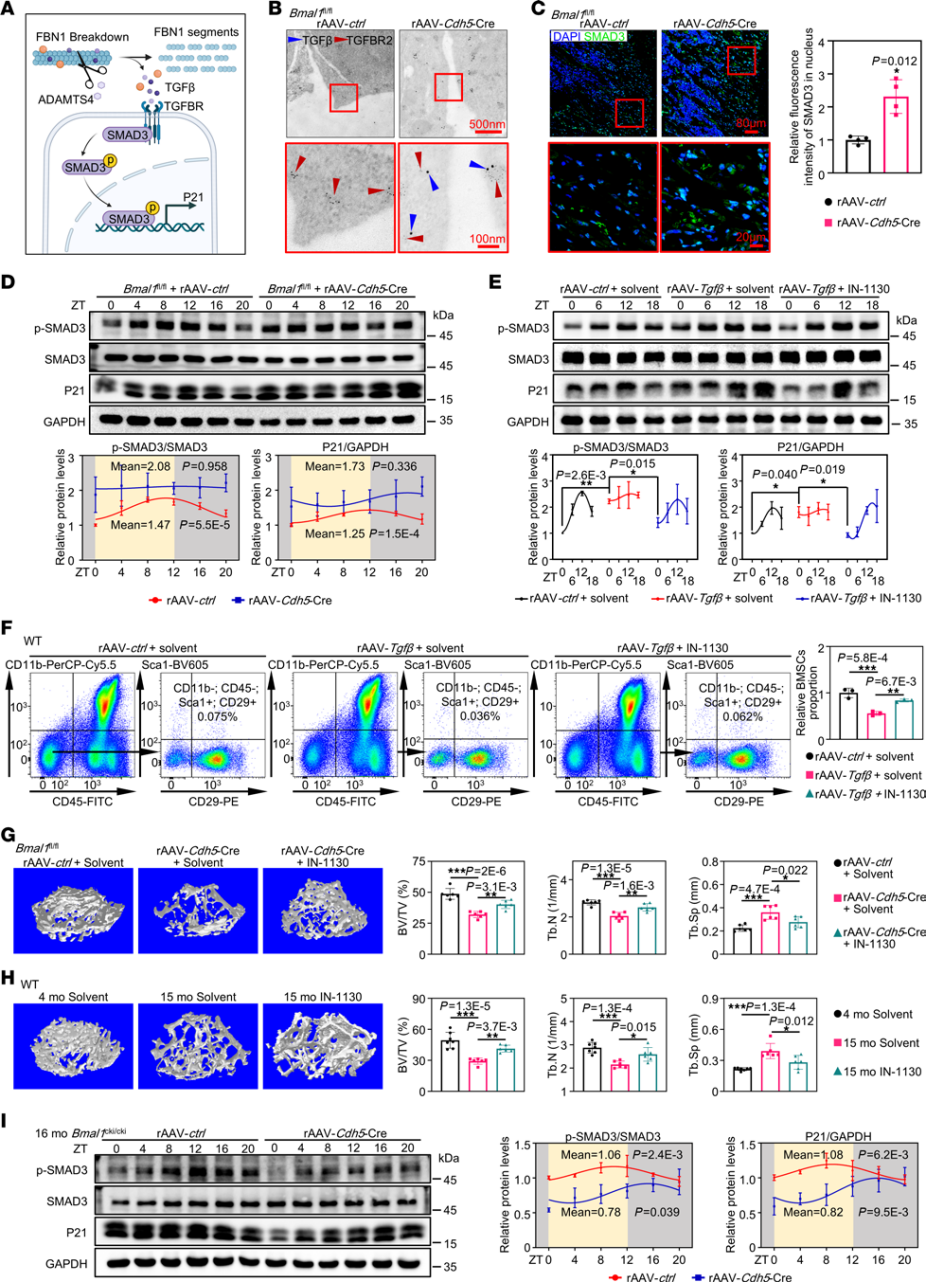
Degradation of FBN1 triggers persistent activation of TGF-β signaling, leading to BMSC exhaustion. (**A**) Schematic diagram showing the ADAMTS4/FBN1/TGF-β/SMAD3/P21 signaling pathway. (**B**) Immunogold-sliver double-labeling of TGF-β and TGF-BR2 in femurs (*n* = 3). Scale bars: 500 nm and 100 nm (enlarged insets). (**C**) Immunofluorescence images of SMAD3 in femurs and quantitative data showing the relative fluorescence intensity of SMAD3 in the nucleus (*n* = 4). Scale bars: 80 μm and 20 μm (enlarged insets). (**D**) Western blot analysis and quantitative data of the levels of p-SMAD3, SMAD3, and P21 in femurs over a 1-day period (*n* = 3). (**E**) Western blot analysis and quantitative data for p-SMAD3/SMAD3 and P21 protein levels in femurs. Each regression curve refers to normalized mean densitometric values of a proteasome protein from the Western blot above. The curves refer to the average trend of diurnal changes in p-SMAD3 or P21 protein levels, which the sine wave model produced (*n* = 3). (**F**) Representative flow cytometry plot and quantitative data showing CD11b^–^CD45^–^Sca1^+^CD29^+^ BMSCs (*n* = 3). (**G**) Representative images of micro-CT reconstruction of femurs, with quantitative data shown. *Bmal1^fl/fl^* mice were injected with rAAV-*ctrl* or rAAV-*Cdh5*-Cre at 3 months of age, and then injected with IN-1130 at 5 months of age for 1 month (*n* = 6). (**H**) Representative images of micro-CT reconstruction of femurs from mice injected with IN-1130 or solvent for 1 month, with quantitative data shown. *n* = 7 (4 mo solvent), *n* = 6 (15 mo solvent, 15 mo IN-1130). (**I**) Western blot analysis of p-SMAD3, SMAD3, and P21 protein levels over a 1-day period in aged femurs treated with rAAV (*n* = 3). Shadow, night; yellow, day; ZT0, 8 am, light turned on. *P < 0.05, **P < 0.01, and ***P < 0.001. Data in **C** were analyzed by unpaired, 2-tailed *t* test with Welch’s correction; data in **D** and **I** were analyzed using DiscoRhythm (cosinor analysis); data in **F**, **G**, and **H** were analyzed by 1-way ANOVA with Tukey’s multiple-comparison test; and data in **E** were analyzed by 2-way ANOVA with multiple comparisons.

Following the degradation of extracellular FBN1, a proportion of TGF-β molecules are released and subsequently bind to receptors situated on the cell surface (53). This leads to the promotion of small mother against decapentaplegic family member 3 (SMAD3) phosphorylation (p-SMAD3), resulting in the activation of *P21* transcription (Figure 6A) (54–56). Through a double-labeling experiment utilizing ISEM, we confirmed that the TGF-β receptors on the cell membranes of endothelial *Bmal1*–deficient mice adhered to more TGF-β than was observed in the control mice (Figure 6B). Consistent with increased TGF-β release, a greater amount of SMAD3 signaling was observed to colocalize with the nucleus, indicating the activation of TGF-β signaling (Figure 6C). Next, we performed Western blot analysis to test whether a decline in endothelial *Bmal1* affects TGF-β/SMAD3 signaling. We observed an oscillation of p-SMAD3 and p21 expression, with the highest expression at zeitgeber time 12 (ZT12), and the oscillation disappeared in endothelial *Bmal1*–deficient mice, with a marked increase in the levels of both p-SMAD3 and p21 (Figure 6D). Subsequently, we tested whether constant activation of TGF-β signaling could mimic the BMSC defects induced by a reduction in endothelial BMAL1 using a model in which mice were injected with rAAV-*TGFB*, and whether restoration of the circadian rhythms of TGF-β/SMAD3 signaling could restore the BMSC defects by administering IN-1130, an inhibitor of TGF-β type 1 receptor (TGF-βR), 1 hour before light exposure (ZT23) every day for 2 weeks. The femurs of mice treated with rAAV-*TGFB* and IN-1130 displayed a reconstruction of the expression rhythms of p-SMAD3 and P21, as demonstrated by Western blotting (Figure 6E). The flow cytometric results further showed that the decrease in BMSCs caused by continuous TGF-β signaling activation was mostly reversed by IN-1130 chrono-treatment (Figure 6F). Both the endothelial *Bmal1*-deficient mice and the aged mice demonstrated a consistent reversal of bone mass loss (Figure 6, G and H). We found that IN-1130 decreased the osteoclast numbers in the aged mice (Supplemental Figure 10A), consistent with the report that TGF-β could promote osteoclast formation (57, 58). Moreover, we showed that upregulation of BMAL1 in aged mice could effectively impede the excessive TGF-β/SMAD3 signaling and diminish the abnormal P21 protein levels in their femurs, restoring the rhythm of the TGF-β/SMAD3 signaling pathway (Figure 6I). These observations suggested that the persistent activation of TGF-β signaling, caused by the degradation of extracellular FBN1 due to the deficiency of BMAL1 in ECs, resulted in the exhaustion of BMSCs and ultimately led to skeletal aging.

### BMAL1 is responsible for regulating TGF-β/SMAD3 signaling and coordinating ADAMTS4 transcription.

**Figure 7:**
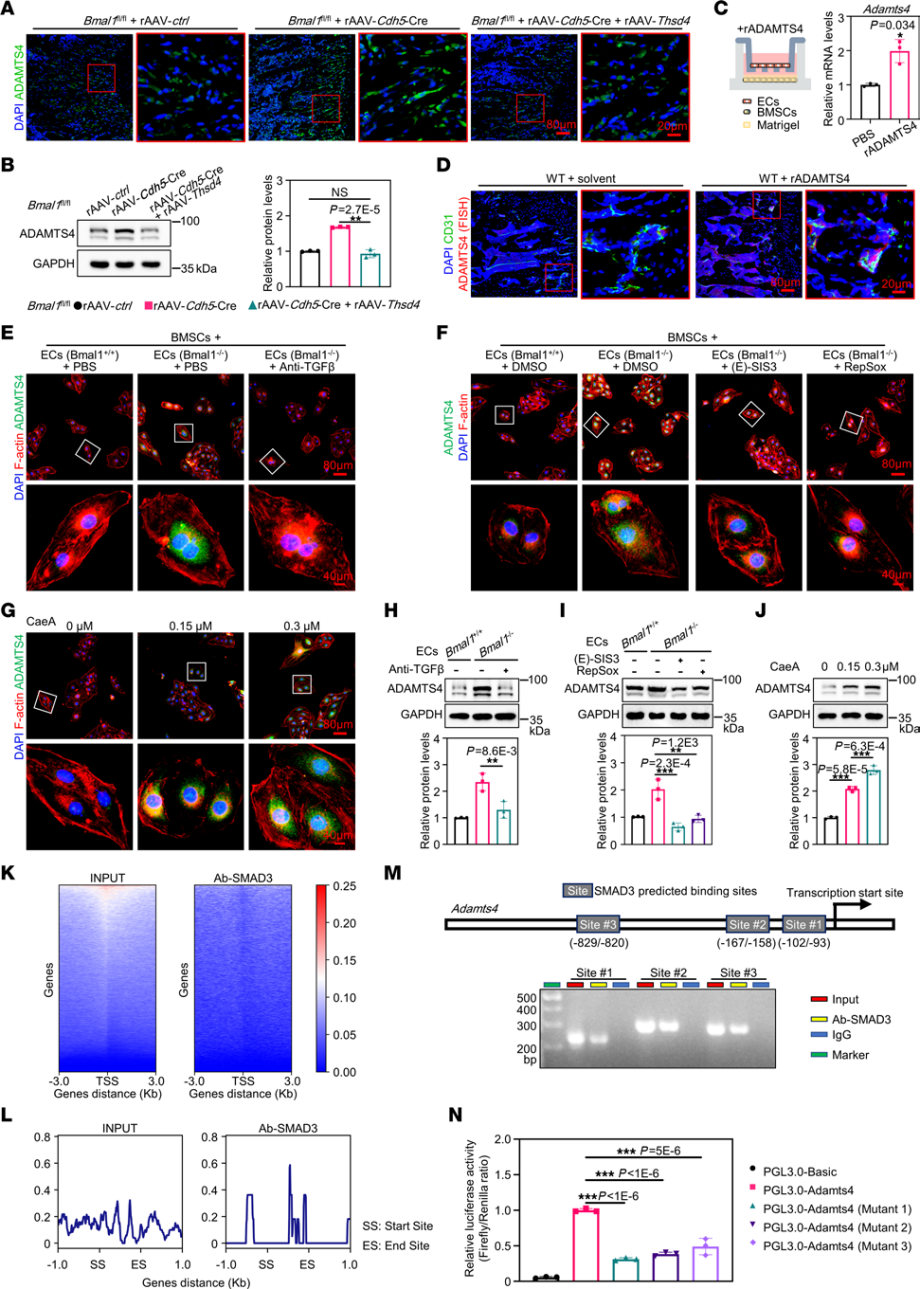
BMAL1 is responsible for regulating TGF-β/SMAD3 signaling and coordinating *Adamts4* transcription. (**A**) Immunofluorescence images of ADAMTS4 expression in femurs of *Bmal1^fl/fl^* mice injected with rAAV-*ctrl*, rAAV-*Cdh5*-Cre or rAAV-*Thsd4*. *Bmal1^fl/fl^* mice were injected with rAAV-*ctrl* or rAAV-*Cdh5*-Cre at 3 months of age and then injected with rAAV-*Thsd4* at 5 months of age (*n* = 3). Scale bars: 80 μm and 20 μm (enlarged insets). (**B**) Western blot analysis of ADAMTS4 protein levels in femurs corresponding to the samples in **A** (*n* = 3). (**C**) Schematic diagram of the ECs and BMSCs indirectly cocultured in Matrigel treated with rADAMTS4. Quantitative data show relative mRNA levels of *Adamts4* in ECs by qPCR (*n* = 3). (**D**) Representative FISH images of ADAMTS4 and Immunofluorescence images of CD31 in femurs from WT mice injected with rADAMTS4 or solvent (*n* = 3). Scale bars: 80 μm and 20 μm (enlarged insets).(**E**–**G**) Representative immunofluorescence images of ADAMTS4 of ECs that were cocultured with BMSCs subjected to the indicated treatment (*n* = 3). Scale bars: 80 μm and 40 μm (enlarged insets). (**H**–**J**) Western blot analysis of ADAMTS4 protein levels in ECs that were cocultured with BMSCs with the indicated treatment and quantitative data (*n* = 3). (**K**) Heatmap showing SMAD3-binding peaks in promoter regions. TSS, transcription start site. (**L**) Read coverage profiles of SMAD3-binding peaks in the promoter of *Adamts4* (from start site [SS] to end site [ES]). (**M**) The transcription factor SMAD3 bound to the *Adamts4* promoter in ECs. ChIP assays were performed using anti-IgG as a negative control (*n* = 3). (**N**) Luciferase reporter assays measuring the activity of the WT or mutated SMAD3-binding site at the *Adamts4* promoter in ECs (*n* = 3). *P < 0.05, **P < 0.01, and ***P < 0.001. Data in **C** were analyzed by unpaired, 2-tailed *t* test with Welch’s correction; data in **B**, **H**, **I**, **J**, and **N** were analyzed by 1-way ANOVA with Tukey’s multiple-comparison test.

To gain a more comprehensive understanding of the function of how ADAMTS4 i regulated by endothelial BMAL1 in bone aging, we undertook an analysis of ADAMTS4 levels in endothelial *Bmal1*–deficient mice (Figure 7, A and B). Injection of rAAV-*Thsd4* into the knees of endothelial *Bmal1*–deficient mice resulted in a notable inhibition of ADAMTS4 expression in femurs (Figure 7, A and B), suggesting that ADAMTS4 might be a downstream target of THSD4. The deficiency of *Bmal1* in ECs caused a reduction in THSD4 expression, impeding the assembly of extracellular FBN1 polymers and thus leading to the release of TGF-β. Therefore, we hypothesized that the increase in ADAMTS4 expression in ECs lacking BMAL1 was contingent on the liberation of TGF-β following the degradation of FBN1. To test this hypothesis, we cocultured BMSCs and ECs indirectly in Matrigel and measured the change in ADAMTS4 expression when FBN1 was degraded by treating Matrigel with the enzyme rADAMTS4 and observed that the transcription of *Adamsts4* in ECs was markedly increased when treated with rADAMTS4 (Figure 7C). Then, we applied an ADAMTS4-specific mRNA probe labeled with Cy3 to conduct a FISH assay to measure *Adamts4* transcription in femurs treated with rADAMTS4 to degrade extracellular FBN1. Transcription of *Adamsts4* in ECs displayed a pattern similar to that seen with the in vitro coculture experiments in Matrigel (Figure 7D). The results indicated that the increase in ADAMTS4 expression in ECs lacking *Bmal1* may work as a form of positive feedback that relied on the degradation of extracellular FBN1.

To ascertain whether the expression of ADAMTS4 can be activated by released TGF-β subsequent to extracellular FBN1 degradation, we used a TGF-β–neutralizing antibody, a TGF-β receptor inhibitor (RepSox), or a SMAD3 phosphorylation inhibitor [(E)-SIS3] to interfere with the indirect coculturing system of ECs and BMSCs. Our results demonstrated that inhibition of TGF-β/SMAD3 signaling could effectively reduce the expression of ADAMTS4, which was elevated in ECs with *Bmal1* KO (Figure 7, E, F, H, and I). Likewise, CaeA, an agonist of the TGF-β/SMAD3 signaling pathway, induced the expression of ADAMTS4 in ECs (Figure 7, G and J). Next, we tested whether ADAMTS4 is a direct target of SMAD3 in ECs by ChIP-Seq, since SMAD3 is a conventional transcription factor, and 1,114 peaks of SMAD3 binding were localized to promoter regions, 2 of which in the promoter of *Adamts4* (Figure 7, K and L). Three binding sites of SMAD3 were detected in the 2 peak regions of *Adamts4* (Figure 7M). Through ChIP–quantitative PCR (ChIP-qPCR) and a luciferase reporter assay using *Admats4* promoter mutants, we confirmed that SMAD3 was capable of binding to the *Adamts4* promoters and activating the transcription of *Adamts4* in ECs (Figure 7, M and N). Taken together, our data demonstrated that endothelial SMAD3 had a regulatory effect on ADAMTS4 expression, which relied on the degradation of extracellular FBN1 and the TGF-β/SMAD3 signaling pathway.

Finally, we explored the role of endothelial BMAL1 in bone aging in female mice. By micro-CT and flow cytometric analysis, we found that bone mass and the number of BMSCs decreased in adult female mice after the depletion of femoral endothelial *Bmal1* (Supplemental Figure 11, A and B). The amount of FBN1 in the extracellular matrix was also slightly reduced (Supplemental Figure 11C). In addition, Western blot results showed that loss of endothelial BMAL1 in female mice resulted in decreased THSD4 expression, increased ADAMTS4 expression, and increased phosphorylation of SMAD3, but no marked increase in P21 (Supplemental Figure 11D). These results suggested that endothelial BMAL1 also plays important roles in female bone aging, but the effects were not as obvious as in male mice. Taken together, these findings highlighted sex differences in the mechanism of bone aging and may help complete the full story of bone aging.

## Discussion

It is well known that stem cell depletion is an important cause of bone loss in men during aging (3, 59, 60). Researchers have noticed that the formation of an inflammatory microenvironment in bone marrow during aging attenuates stem cell proliferation and osteogenic differentiation, leading to age-related osteoporosis (61). Factors affecting the bone marrow microenvironment during aging encompass reduced secretion of hormones, such as growth hormone, cortisol, and melatonin (11, 12, 62). Deng et al. revealed that parathyroid hormone can partially prevent skeletal aging and osteoporosis by activating histone lysine demethylase 4B, an epigenetic factor, in stem cells (6). But that does not fully address how hormones cause stem cell depletion. Bone marrow vascular ECs serve as a crucial conduit for hormones to access bone tissue. Here, we found that ECs participated in orchestrating the skeletal aging process in male mice. ECs with BMAL1 scarcity induced a severe decrease in the proliferation of BMSCs, reduced osteogenic activity in BMSCs, and mildly increased osteoclasts in bone tissue, ultimately resulting in senile bone loss. Taken together, one can hypothesize that the fundamental mechanism of ECs mediating the aging signals from the body to bone tissue may play vital roles in natural aging-related bone loss in men.

One of the most prominent features of male bone aging is diminished osteogenic activity. The correlation between blood vessels in bone and osteogenesis is widely acknowledged (63). ECs secrete a plethora of signaling molecules, including BMP2, PDGF-BB, VEGF, etc., in a paracrine manner to modulate osteogenic mineralization(64–68). Our previous research has also demonstrated that ECs are capable of regulating the osteogenic differentiation of BMSCs through juxtacrine signaling during bone regeneration (69). The extracellular vesicle is another marked pathway through which ECs modulate osteogenic activity (41, 70, 71). Our previous studies have also indicated that the ECM has an effect on the classification and function of ECs (72). The findings of this study suggested an interaction between ECs and the ECM, and the complex mechanism of this interaction needs to be further explored. Bone ECM is a crucial component that plays a vital role in regulating the activities of osteoblasts and osteoclasts throughout various stages of bone development, regeneration, and aging (73–77), all of which are facilitated by cytokines present within the ECM (73–77). Among various cytokines in the ECM, TGF-β is a vital regulatory factor that modulates the progression of BMSC proliferation and differentiation (55, 56, 78–81). TGF-β is stored in or released from the ECM, depending on the status of extracellular microfibrils assembled by FBN1 (47). We identified a FBN1 protein–cleaving enzyme, ADAMTS4, that worked with THSD4 to maintain the balance of FBN1 degradation. We found that FBN1 breakdown resulted in BMSC depletion, which was consistent with the reports that FBN1 KO impairs bone development and remodeling (82–84). On the other hand, enhanced osteoclast activity is another prominent feature of bone aging. But the number of osteoclasts in this study only slightly increased when endothelial BMAL1 was absent. It could be explained that TGF-β–promoted osteoclast differentiation was partially inhibited by the FBN1 cleavage products rF23 and rFBN1-N (57, 58, 85). On the basis of these data, it could be inferred that ECs may act as the primary drivers of extracellular microfibril remodeling, which in turn determines the fate of BMSCs and osteoclast precursor cells and thus affects bone metabolism. Through these studies, we identified a molecular network among ECs, ECM, BMSCs, and osteoclasts that revealed a fundamental association between ECs and bone metabolism.

Recent studies illuminated the interplay between different types of ECs and osteoblast lineage cells (13, 68, 86–91) and categorized ECs into 3 subtypes: type E, type H, and type L. Different subtypes of ECs have different effects on the promotion of osteogenic activity. Here, we showed that the abundance of endothelial BMAL1 indicated their ability to promote osteogenesis. We observed that as aging occurred, the abundant expression of endothelial BMAL1 decreased, resulting in a reduced ability to promote osteogenesis. From the perspective of diurnal variation controlled by BMAL1, ECs affected the rhythms of bone homeostasis by rhythmically regulating the activation of the TGF-β/SMAD3 signaling pathway. In young individuals, the rhythmic oscillation of BMAL1 expression was stronger, and its expression abundance was higher (92, 93). We found that rhythmic oscillations in bone metabolic activity were well maintained when endothelial BMAL1 expression had a strong circadian rhythm, as reflected in the diurnal oscillation of the phosphorylation levels of SMAD3 and expression of the downstream genes of TGF-β/SMAD3 signaling. In the absence of endothelial BMAL1, TGF-β/SMAD3 signaling was continuously activated and lost its circadian rhythm, persistently suppressing the proliferation of BMSCs, and ultimately leading to BMSC depletion. Our finding was in line with the existing literature indicating that excessive TGF-β signaling in bone results in suppression of BMSC proliferation, which is associated with a reduction in bone density and stunted growth (54, 94, 95). Collectively, our findings indicated that disrupting the body’s natural circadian rhythms may have deleterious effects, akin to the detrimental consequences of excessive water.

BMAL1 is a transcription factor that promotes osteogenic activity and has been well studied in BMSCs, osteoclasts, and osteoblasts (37, 96–99). It has been reported that osteoclast-specific deletion of *Bmal1* does not affect bone mass (100). However, another study reported that mice with osteoclast-specific *Bmal1* deletion had higher bone mass than WT mice (101). As for BMSCs, *Bmal1* deficiency in mesenchymal cells resulted in a reduction of approximately 20% in bone mass (100). Our research revealed that the elimination of *Bmal1* in ECs led to a decrease in bone mass of more than 40% (Figure 2D), which more closely resembled the bone phenotype of mice with *Bmal1* global KO. These data reinforced the notion that, in the regulation of bone metabolism by BMAL1, ECs play a more crucial role than other cell types, which may be due to the gated role of ECs in mediating aging signals to bone tissue. We found that the decline of endothelial BMAL1 due to aging played a marked role in the progression of skeletal aging. The effective alleviation of bone aging and restoration of bone mass in aged mice could be achieved by restoring BMAL1 in ECs, suggesting that small-molecule agonists of BMAL1 could be potential candidates for clinical treatment of age-related osteoporosis.

Sex differences are present in the developmental process and the molecular mechanisms of bone aging. For women, estrogen is a key factor in maintaining bone homeostasis. In the female menopause, estrogen levels drop sharply, causing osteoclasts to become active, and overactivated osteoclasts promote the enhancement of osteogenic activity (10, 102). Estradiol, a well-known and powerful estrogen, inhibits osteoclast formation and bone resorption activity by downregulating the NF-κB pathway and regulating RANKL binding (103–105). In addition, estradiol also promotes osteoblast and new bone formation by upregulating the expression of B cell lymphoma 2 (BCL-2), alkaline phosphatase, and bone-specific alkaline phosphatase (106–108). Our results suggested that in elderly males, a decline in endothelial BMAL1 expression accelerated bone aging by depleting BMSCs and increasing osteoclastic activity. And we devised a method that revealed a more clearly defined mechanism in male mice than female mice that the continuous release of TGF-β from structurally degraded FBN1 in bone matrix was a vital factor driving bone aging that was mainly triggered by the aging-related imbalance of THSD4 and ADAMTS4 mediated by endothelial BMAL1. Our findings highlighted sex differences in the mechanism of bone aging and may help complete the full picture of this phenomenon.

An aging hallmark needs to meet 3 characteristics, including age-related alterations, the possibility of accelerating aging, and the opportunity to decelerate, halt, or reverse aging by therapeutic interventions on the hallmark (109). THSD4 and ADAMTS4 exhibited age-related changes that accompanied the aging process, and aging could be accelerated or alleviated by regulating these 2 proteases, suggesting that they were tightly related to aging and could be used to predict premature aging and age reversal phenotypes. FBN1 is a necessary ECM protein that serves as a major structural component of ECM and attaches a large amount of TGF-β, affecting the process of cellular senescence (110, 111). Stem cell depletion is already a well-established indicator of aging (109). In aged bone tissue, the regeneration and differentiation of BMSCs are damaged, and BMSCs appear in a replicative senescence state or even exhausted, leading to defects in the renewal of osteoblast lineage cells (112, 113). Taken together, our findings suggested that the status of THSD4, ADAMTS4, BMSCs, and FBN1 deeply affected the occurrence and development of bone aging, providing a target for predicting and reversing bone aging.

## Methods

### Sex as a biological variable.

Our study examined male and female animals, and similar findings are reported for both sexes.

### Statistics.

All data are displayed as the mean ± SD. GraphPad Prism 9.0.2 (GraphPad Software) was used for the statistical analyses. Figure 4I and Figure 6D were analyzed using DiscoRhythm (https://mcarlucci.shinyapps.io/discorhythm/). Data were evaluated by unpaired, 2-tailed *t* test or by 1-way ANOVA, Tukey’s multiple-comparison test. A *P* value of less than 0.05 was considered significant. All source numerical data are provided in the Supporting Data Values file.

### Study approval.

All animal studies were performed in mice and approved by the Huazhong University of Science and Technology Laboratory Animal Centre and Use Committee (IACUC number: 3293).

### Data availability.

The analytic methods, and study materials are described in full in the Supplemental Methods. A Supporting Data Values file is available online as supplemental material. All gel data and IBs in this study are reported in the full, unedited gel file available in the supplement with full annotations. mRNA-Seq and ChIP-Seq data reported in this publication have been submitted to National Center for Biotechnology Information’s Sequence Read Archive (SRA) database (BioProject numbers: PRJNA1165254 and PRJNA1165972). All custom code used in this study is available from the corresponding author upon reasonable request.

## Author contributions

YY, QT, and LC were responsible for the study concept and design. YY, JY, SG, YZ ,and ZH performed experiments. YY, QT, YS, XZ, SY, GC, JS, and LZ acquired, analyzed, and interpreted data. YY and QT drafted the manuscript. LC provided critical revision of the manuscript and important intellectual content. LC supervised the study. LC and QT acquired funding for the work. All authors reviewed the manuscript.

## Supplementary Material

Supplemental data

Unedited blot and gel images

Supplemental tables 1-5

Supporting data values

## Version 1 12/16/2024

Electronic publication
