# Supplementary material for: Endothelial BMAL1 decline during aging leads to bone loss by destabilizing extracellular fibrillin-1
Source: J Clin Invest. 2024 Dec 16;134(24):e176660. doi: 10.1172/JCI176660 (PMC11645155; doi:10.1172/JCI176660)
Supplement: Supplemental data [file jci-134-176660-s072.pdf]

Endothelial BMAL1 decline during aging leads to bone loss by destabilizing extracellular Fibrillin-1

Ying Yin[1, 2, 3], Qingming Tang[1, 2, 3], Jingxi Yang[1, 2, 3], Shiqi Gui[1, 2, 3], Yifan Zhang[1, 2, 3], Yufeng Shen[1, 2, 3], Xin Zhou[1, 2, 3], Shaoling Yu[1, 2, 3], Guangjin Chen[1, 2, 3], Jiwei Sun[1, 2, 3], Zhenshuo Han[1, 2, 3], Luoying Zhang[4], and Lili Chen[1, 2, 3]

[1]Department of Stomatology, Union Hospital, Tongji Medical College, Huazhong University of Science and Technology, Wuhan 430022, China

[2]School of Stomatology, Tongji Medical College, Huazhong University of Science and Technology, Wuhan 430030, China

[3]Hubei Province Key Laboratory of Oral and Maxillofacial Development and Regeneration, Wuhan 430022, China

[4]Key Laboratory of Molecular Biophysics of Ministry of Education, College of Life Science and Technology, Huazhong University of Science and Technology, Wuhan 430074, China

Authorship notes: Ying Yin and Qingming Tang contributed equally to this work.

Address correspondence to: Lili Chen, Department of Stomatology, Union Hospital, Tongji Medical College, Huazhong University of Science and Technology, 1277 Jiefang Avenue, Wuhan 430022, China. Phone: +86-27-85726949; Email: chenlili1030@hust.edu.cn.

Conflict of interest: The authors have declared that no conflict of interest exists.

Supplementary Figures and Figure Legends

Figure S1, related to Figure 1.

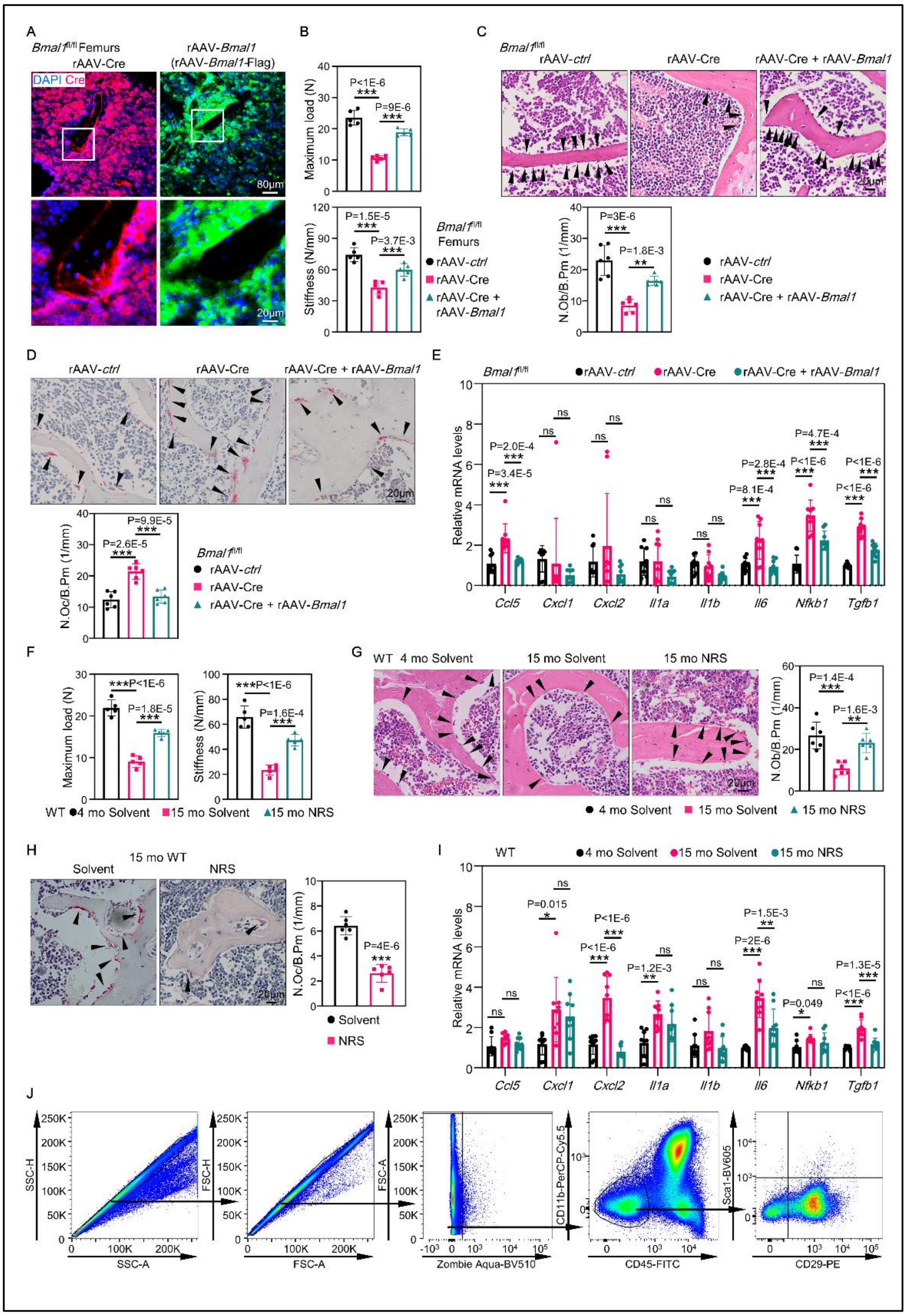

(**A**) Immunofluorescence of Cre or FLAG in femurs. n=3. (**B**) Maximum compressive load and stiffness of femurs corresponding to the samples in **Figure 1D**. n=5. (**C**) Representative images of H&E staining of femurs corresponding to the samples in

Figure 1D with quantitative data below. Black arrowheads point to osteoblasts. n=6. (**D**) Representative images of TRAP staining of femurs corresponding to the samples in **Figure 1D** with quantitative data below. Black arrowheads point to osteoclasts. n=6. (**E**) Quantitation data showing relative mRNA levels of *Ccl5*, *Cxcl1*, *Cxcl2*, *Il1a*, *Il1b*, *Il6*, *Nfkb1*, and *Tgfb1* of femoral bone marrow corresponding to the samples in **Figure 1D** by qRT-PCR. n=3. (**F**) Maximum compressive load and stiffness of femurs corresponding to the samples in **Figure 1J**. **n**=5. (**G**) Representative images of H&E staining of femurs corresponding to the samples in **Figure 1J** with quantitative data at right. Black arrowheads point to osteoblasts. n=6. (**H**) Representative images of TRAP staining of femurs corresponding to the samples in **Figure 1J** with quantitative data at right. Black arrowheads point to osteoclasts. n=6. (**I**) Quantitation data showing relative mRNA levels of *Ccl5*, *Cxcl1*, *Cxcl2*, *Il1a*, *Il1b*, *Il6*, *Nfkb1*, and *Tgfb1* of femoral bone marrow corresponding to the samples in **Figure 1J** by qRT-PCR. n=3. (**J**) Representative images for FACS identifying BMSCs. Cells were first gated by forward scatter (FSC) and side scatter (SSC) to remove doublets. Live cells were identified by negative gating for Zombie AquaMuSCs. All flow cytometry data in this study were identified as living cells using the above method before identification. BMSCs were identified by CD11b-;CD45-;Sca1+;CD29+. *$p < 0.05$, **$p < 0.01$, ***$p < 0.001$, ns, nonsignificant *P* value. **H**, Data were analyzed using a unpaired t-test with Welch's correction. **B**, **C**, **D**, **E**, **F**, **G**, **I**, Data were analyzed using One-way ANOVA, Tukey's multiple comparisons test.

Figure S2, related to Figure 2.

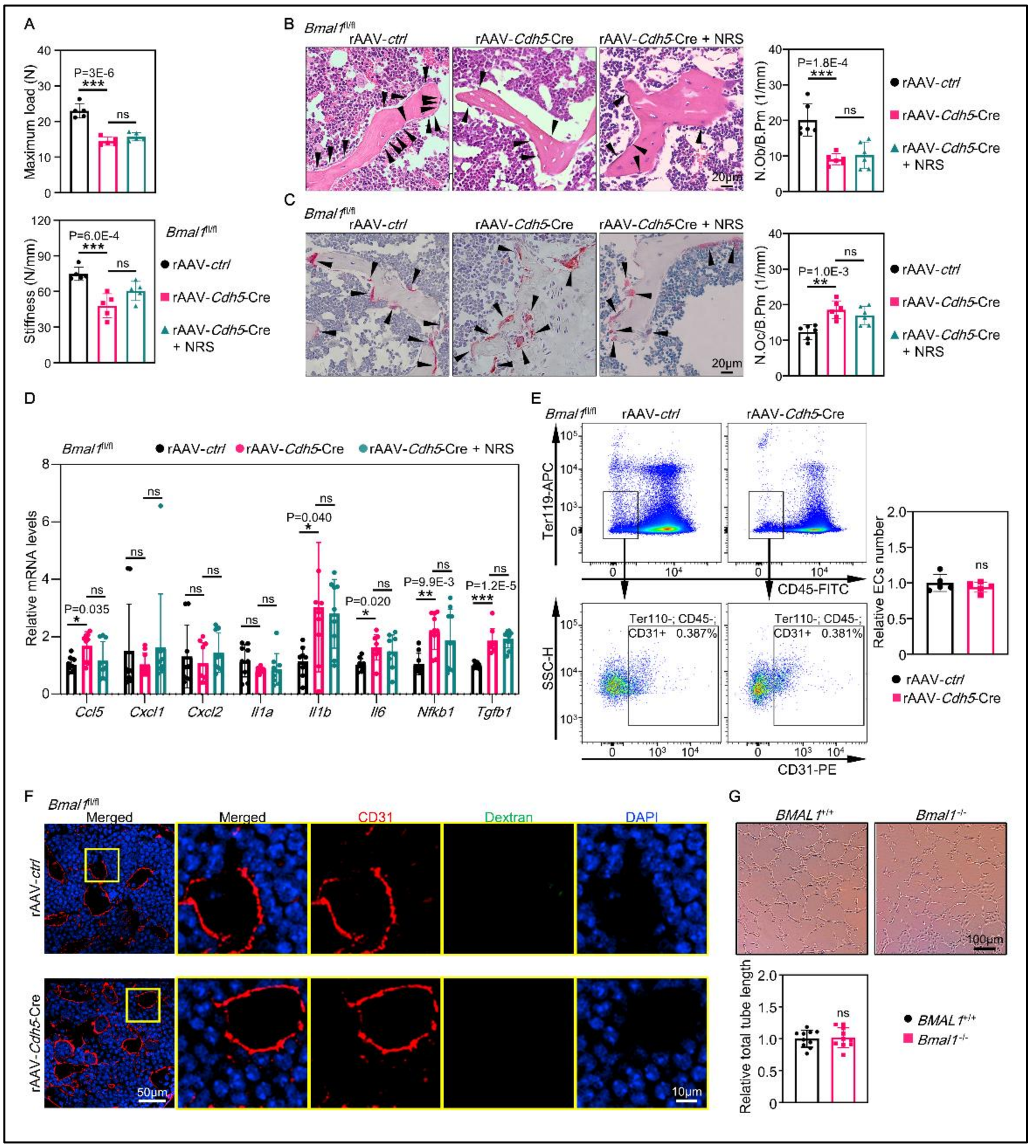

(**A**) Maximum compressive load and stiffness of femurs corresponding to the samples in **Figure 2E**. n=5. (**B**) Representative images of H&E staining of femurs corresponding to the samples in **Figure 2E** with quantitative data at right. Black arrowheads point to osteoblasts. n=6. (**C**) Representative images of TRAP staining of femurs corresponding to the samples in **Figure 2E** with quantitative data at right. Black arrowheads point to osteoclasts. n=6. (**D**) Quantitation data showing relative mRNA levels of *Ccl5*, *Cxcl1*, *Cxcl2*, *Il1a*, *Il1b*, *Il6*, *Nfkb1*, and *Tgfb1* of femoral bone marrow corresponding to the samples in **Figure 2E** by qRT-PCR. n=3. (**E**) Representative flow cytometry analysis for the number of Ter119^{-} CD45^{-} CD31^{+} vascular endothelial cell population in the bone marrow of mice injected with rAAV-*ctrl*, or rAAV-*Cdh5*-Cre, with quantitative data at right. n=5. (**F**) Immunofluorescence of CD31 and Dextran-FITC in femurs of mice injected with rAAV-*ctrl*, or rAAV-*Cdh5*-Cre. (**G**) Representative images of tube formation at 6 h after ECs were seeded on Matrigel. Total tube length were quantified using Image Pro Plus software. *p < 0.05, **p < 0.01,

***p < 0.001, ns, nonsignificant *P* value. **E**, **G**, Data were analyzed using a unpaired t-test with Welch's correction. **A**, **B**, **C**, **D**, Data were analyzed using One-way ANOVA, Tukey's multiple comparisons test.

Figure S3, related to Figure 2.

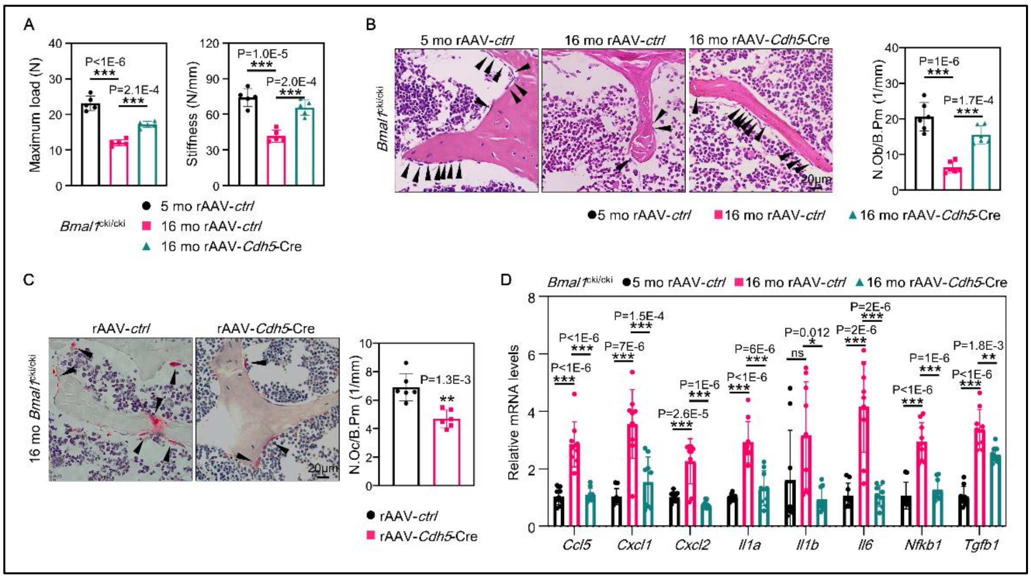

(**A**) Maximum compressive load and stiffness of femurs corresponding to the samples in **Figure 2I**. n=5. (**B**) Representative images of H&E staining of femurs corresponding to the samples in **Figure 2I** with quantitative data at right. Black arrowheads point to osteoblasts. n=6. (**C**) Representative images of TRAP staining of femurs corresponding to the samples in **Figure 2I** with quantitative data at right. Black arrowheads point to osteoclasts. n=6. (**D**) Quantitation data showing relative mRNA levels of *Ccl5*, *Cxcl1*, *Cxcl2*, *Il1a*, *Il1b*, *Il6*, *Nfkb1*, and *Tgfb1* of femoral bone marrow corresponding to the samples in **Figure 2I** by qRT-PCR. n=3. *$p < 0.05$, **$p < 0.01$, ***$p < 0.001$. **C**, Data were analyzed using a unpaired t-test with Welch's correction. **A**, **B**, **D**, Data were analyzed using One-way ANOVA, Tukey's multiple comparisons test.

Figure S4, related to Figure 3.

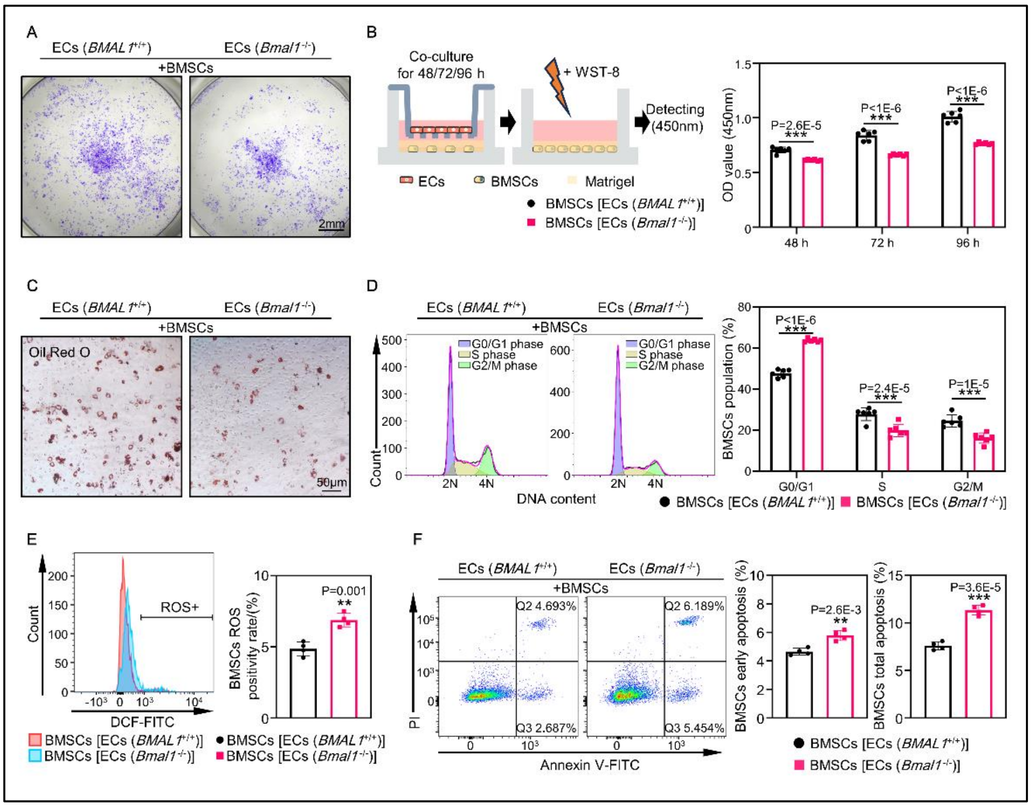

(**A**) Representative clone formation assays of BMSCs indirectly co-cultured with ECs. ECs were sorted from the *Bmal1*$^{fl/fl}$ mice injected with rAAV-*ctrl* or rAAV-*Cdh5*-Cre. n=3. (**B**) Schematic diagram of the CCK8 assays. BMSCs co-cultured with ECs for 48, 72, and 96 h were examined by CCK8 assays. ECs were sorted from the *Bmal1*$^{fl/fl}$ mice injected with rAAV-*ctrl* or rAAV-*Cdh5*-Cre. n=6. (**C**) Representative oil red O staining of BMSCs co-cultured with ECs corresponding to the samples in **A**. n=3. (**D**) Cell-cycle phases were determined by flow cytometry of BMSCs co-cultured with ECs corresponding to the samples in **A**. n=6. (**E**) ROS levels were determined by flow cytometry of BMSCs co-cultured with ECs, corresponding to the samples in **A**. n=4. (**F**) Cell apoptosis was evaluated by flow cytometry of BMSCs stained with Annexin V and PI. co-cultured with ECs, corresponding to the samples in **A**. n=4. *$p < 0.05$, **$p < 0.01$, ***$p < 0.001$. **E**, **F**, Data were analyzed using a unpaired t-test with Welch's correction. **B**, **D**, Data were analyzed using two-way ANOVA, multiple comparisons.

Figure S5, related to Figure 4.

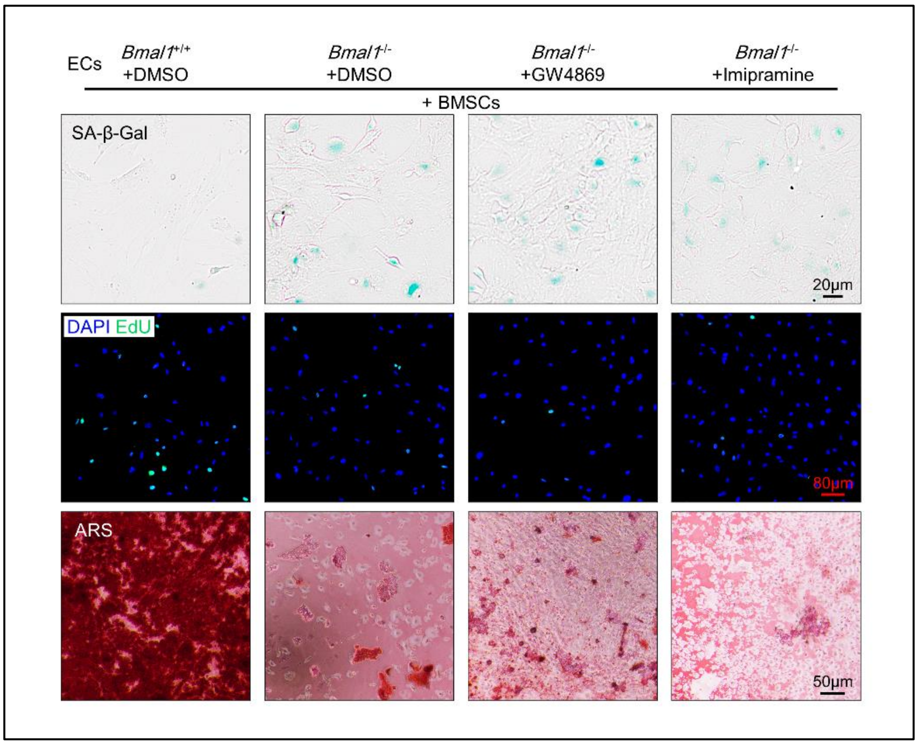

Representative SA-β-Gal staining (blue), EdU immunofluorescence, and ARS staining of BMSCs which co-cultured with ECs at indicated treatment. n=3.

Figure S6, related to Figure 4.

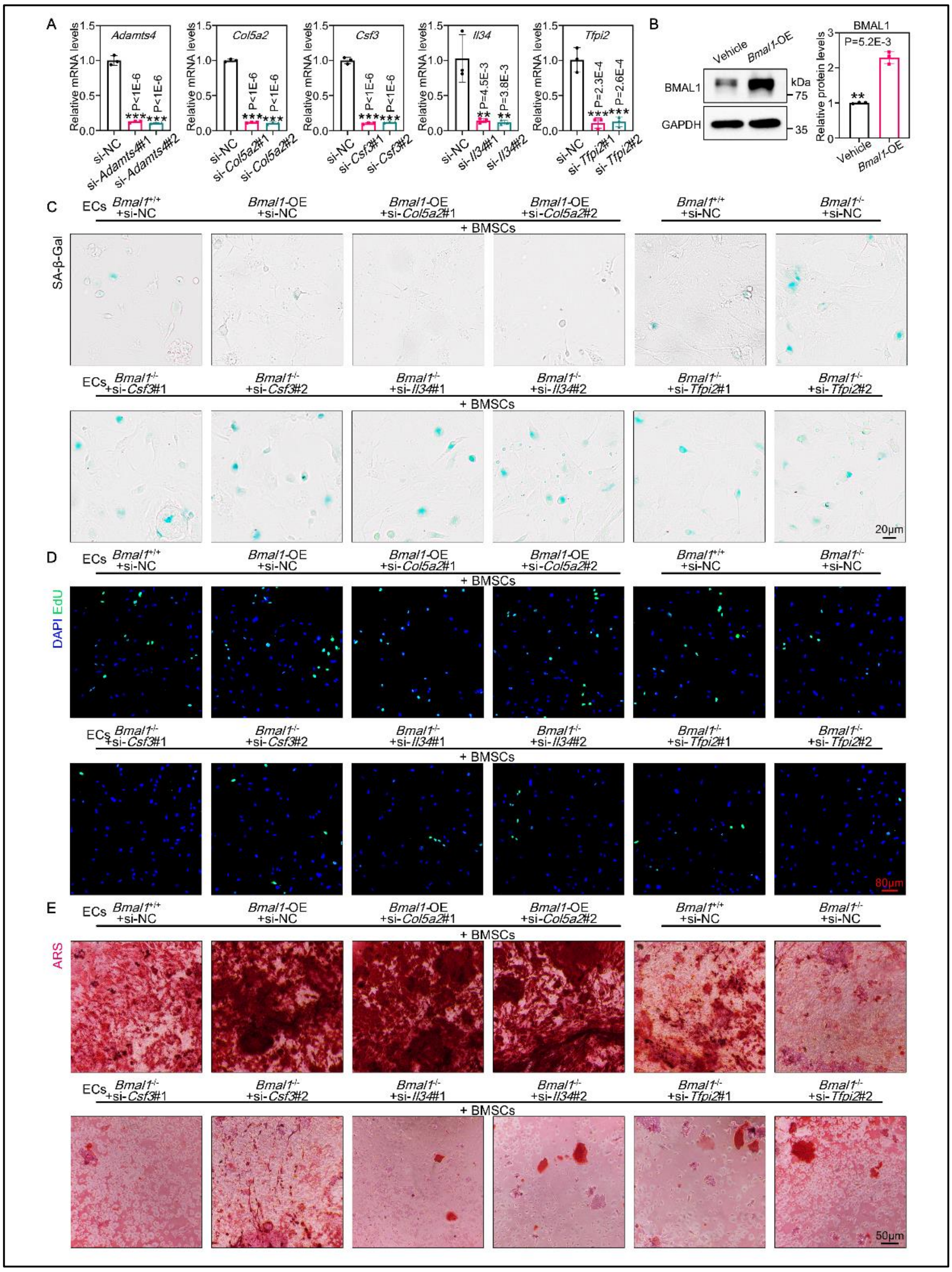

(**A**) *Adamts4*, *Col5a2*, *Csf3*, *Il34*, and *Tfpi2* knockdown were assessed by qRT-PCR. n=3. (**B**) THSD4 overexpression was assessed by western blot assays. n=3. (**C-E**) Representative SA-βGal staining (blue), EdU immunofluorescence, and ARS staining of BMSCs which co-cultured with ECs at indicated treatment. n=3. *p < 0.05, **p < 0.01, ***p < 0.001. **A**, Data were analyzed using One-way ANOVA, Tukey's multiple comparisons test. **B**, Data were analyzed using a unpaired t-test with Welch's correction.

Figure S7, related to Figure 4.

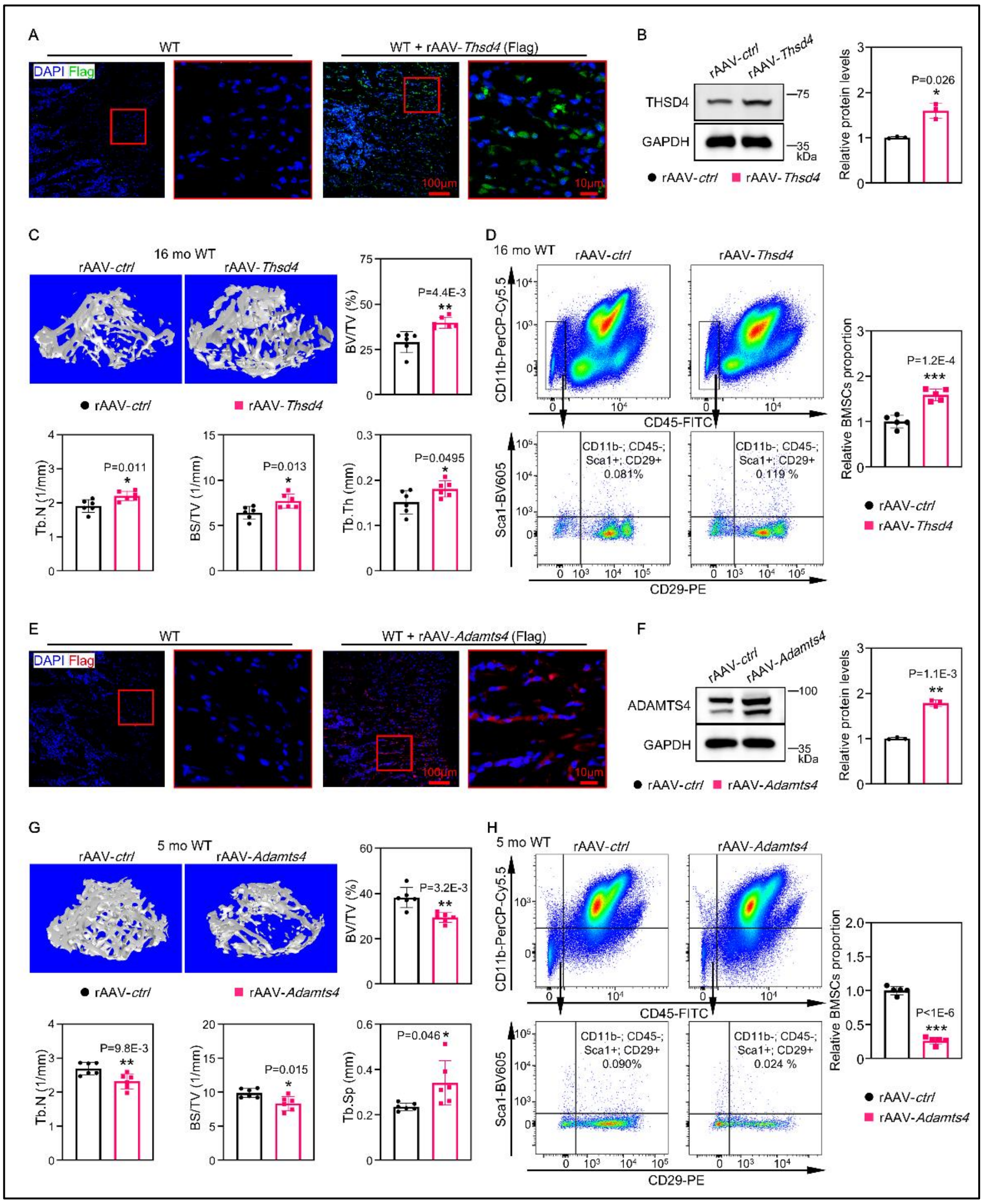

(**A**) Immunofluorescence of Flag in femurs of mice injected with rAAV-*Thsd4*. n=3. (**B**) Western blot analysis of the levels of THSD4 at indicated time in femurs of 16-month-old mice injected rAAV-*ctrl*, or rAAV-*Thsd4* two months ago, with quantitative data at right. n=3. (**C**) Representative images of micro-CT reconstruction of femurs corresponding to the samples in **B**, with quantitative data. n=6. (**D**) Representative flow cytometry plot showing CD11b$^-$;CD45$^-$;Sca1$^+$;CD29$^+$ BMSCs in bone marrow corresponding to the samples in **B**, with quantitative data at right. n=5. (**E**) Immunofluorescence of Flag in femurs of mice injected with rAAV-*Adamts4*. n=3. (**F**) Western blot analysis of the levels of THSD4 at indicated time in femurs of 5-month-old mice injected rAAV-*ctrl*, or rAAV- *Adamts4* two months ago, with quantitative data at right. n=3. (**G**) Representative images of micro-CT reconstruction of femurs corresponding to the samples in **F**, with quantitative data. n=6. (**H**) Representative flow

cytometry plot showing $CD11b^-$;$CD45^-$;$Sca1^+$;$CD29^+$ BMSCs in bone marrow corresponding to the samples in **F**, with quantitative data at right. n=5. *$p < 0.05$, **$p < 0.01$, ***$p < 0.001$. **B**, **C**, **D**, **F**, **G**, **H**, Data were analyzed using a unpaired t-test with Welch's correction.

Figure S8, related to Figure 4.

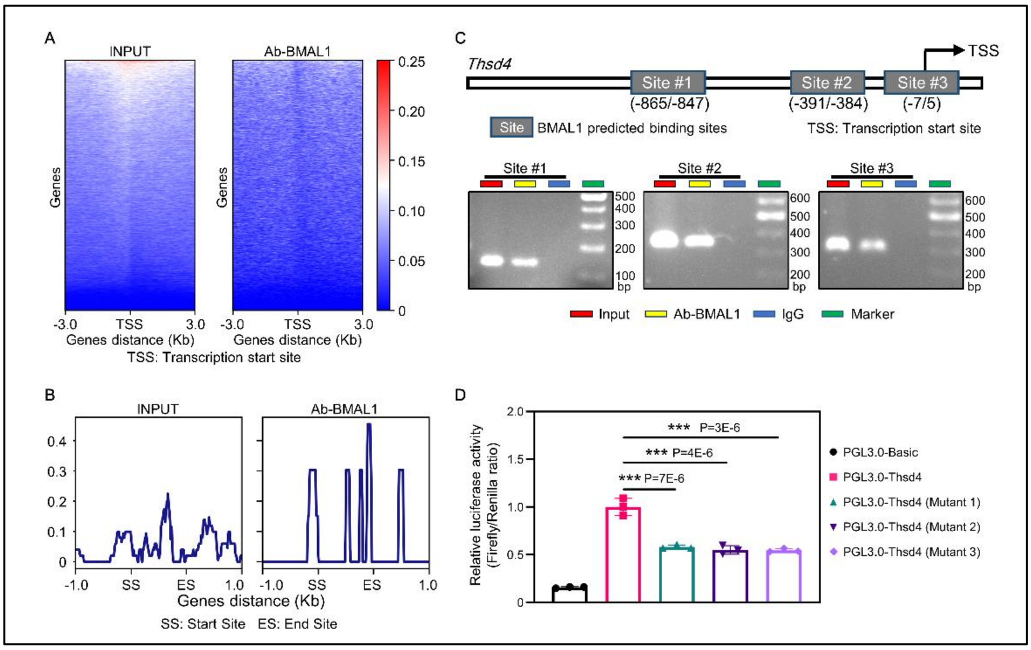

(**A**) Heatmap showing BMAL1-binding peaks in promoter regions. (**B**) Read coverage profiles of BMAL1-binding peaks in the promoter of *Thsd4* (from SS to ES). (**C**) The transcription factor BMAL1 bound to the *Thsd4* promoter in BMECs. Chromatin immunoprecipitation assays were performed using anti-IgG as a negative control. n=3. (**D**) Luciferase reporter assays measuring the activities of the wild-type or mutated BMAL1-binding site at the *Thsd4* promoter in BMECs. n=3. $^{*}p < 0.05$, $^{**}p < 0.01$, $^{***}p < 0.001$. **D**, Data were analyzed using One-way ANOVA, Tukey's multiple comparisons test.

Figure S9, related to Figure 5.

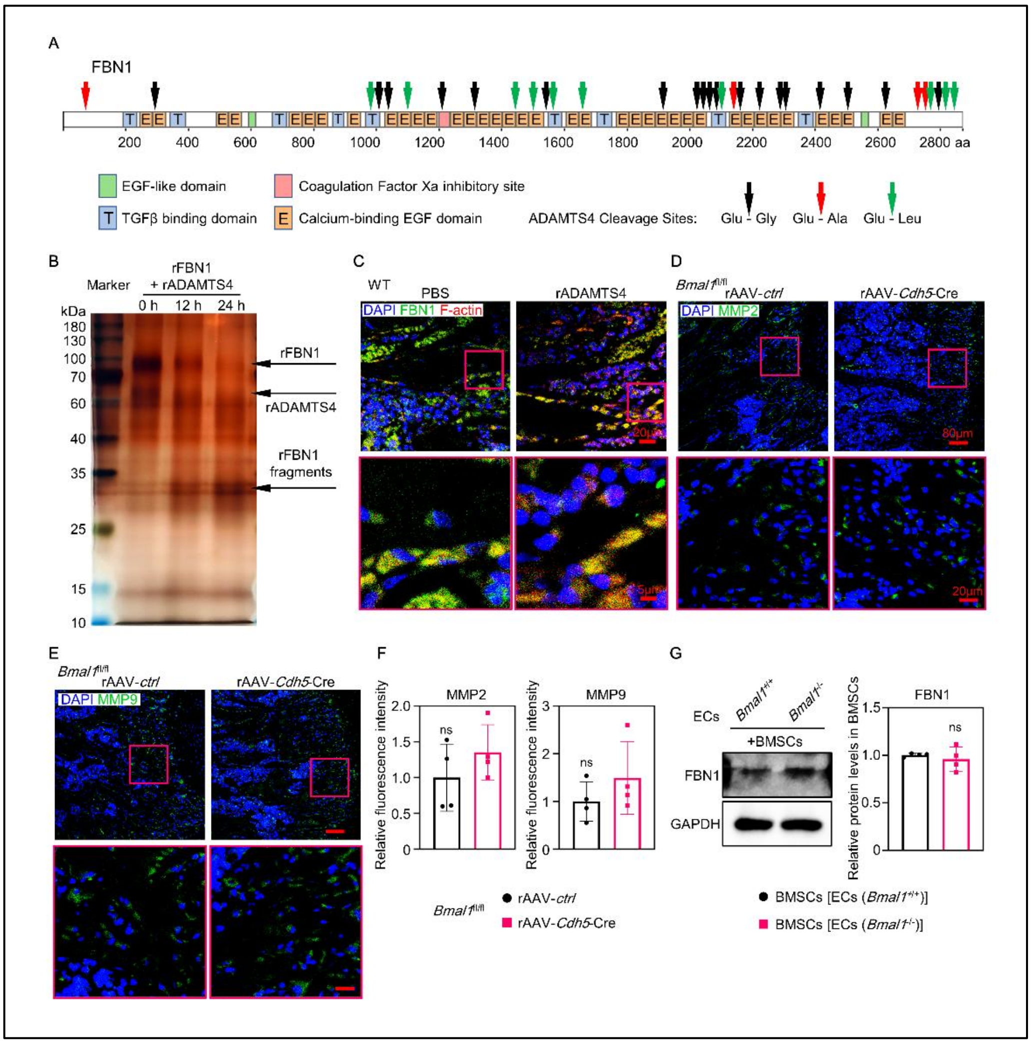

(**A**) Schematic diagram of FBN1 domain organization which indicates the location of the scissile bonds (black, red, and green arrow) that were identified by ADAMTS4. (**B**) Silver stained SDS-PAGE gel of recombinant FBN1 incubated with recombinant ADAMTS4 (rADAMTS4) for 0, 12, and 24 h. n=3. (**C**) Immunofluorescence of Fibrillin-1 (FBN1) and F-actin in femurs of WT mice injected with PBS or rADAMTS4. n=3. (**D** and **E**.) Immunofluorescence of MMP2 and MMP9 in femurs of *Bmal1*$^{fl/fl}$ mice injected with rAAV-*ctrl* or rAAV-*Cdh5*-Cre. n=4. (**F**) Quantitative data of MMP2 and MMP9 fluorescence intensity of **D** and **E**. n=4. (**G**) Western blot analysis of the levels of FBN1 in BMSCs which were co-cultured with ECs sorted from femoral bone marrow of *Bmal1*$^{fl/fl}$ mice injected with rAAV-*ctrl* or rAAV-*Cdh5*-Cre, with quantitative data at right. n=3. ns, nonsignificant *P* value. **F**, **G**, Data were analyzed using a unpaired t-test with Welch's correction.

Figure S10, related to Figure 6.

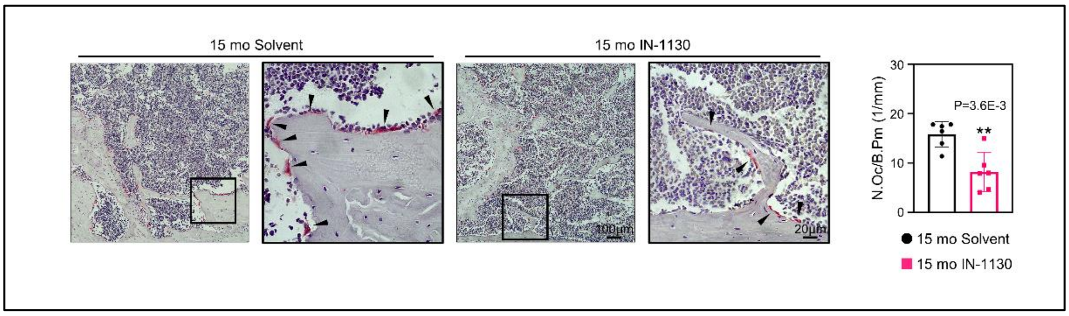

Representative images of TRAP staining of femurs of 14-month-old mice injected with IN-1130 for a month, with quantitative data at right. Black arrowheads point to osteoclasts. n=3. **$p < 0.01$. Data were analyzed using a unpaired t-test with Welch's correction.

Figure S11, related to Figure 7.

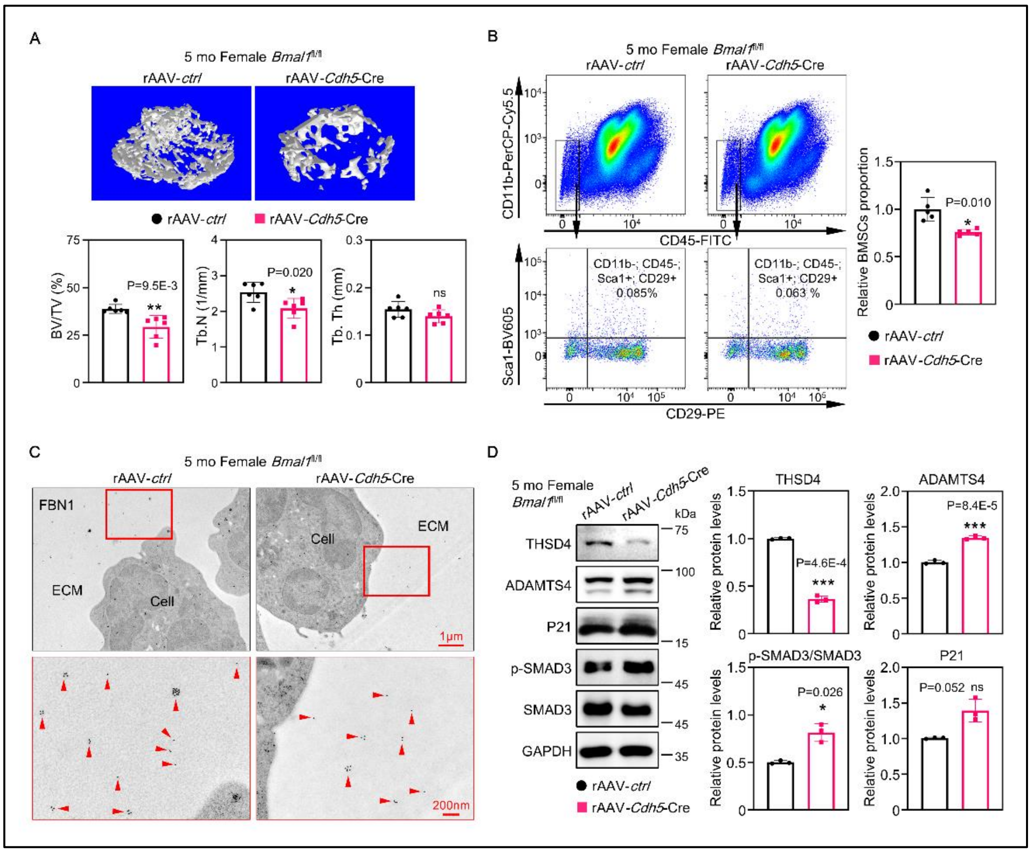

(**A**) Representative images of micro-CT reconstruction of femurs from female 5-month-old *Bmal1*$^{fl/fl}$ mice injected with rAAV-*ctrl* or rAAV-*Cdh5*-Cre two months ago, with quantitative data below. n=6. (**B**) Representative flow cytometry plot showing CD11b^{-};CD45^{-};Sca1^{+};CD29^{+} BMSCs in bone marrow corresponding to the samples in **A**, with quantitative data at right. n=5. (**C**) Immunogold labeling of FBN1 in femurs corresponding to the samples in **A**. n=4. (**D**) Western blot analysis of the levels of THSD4, ADAMTS4, P21, p-SMAD3, and SMAD3 at indicated time in femurs corresponding to the samples in **A**, with quantitative data at right. n=3. *$p < 0.05$, **$p < 0.01$, ***$p < 0.001$, ns, nonsignificant *P* value. **A**, **B**, **D**, Data were analyzed using a unpaired t-test with Welch's correction.

Supplemental Methods

Mice

Bmal1$^{fl/fl}$ mice were kindly provided by Dr. Ying Xu (Soochow University, Jiangsu, China), and *Bmal1*$^{cki/cki}$ mice were purchased from Cyagen (Suzhou, China). All lines were based on a C57BL/6J mouse genetic background. The genotypes of the bred mice were validated by detecting DNA from the scales of the tails of mice. All wild type mice were purchased from the Beijing HFK Bioscience (Beijing, China). Mice were placed randomly under either LD12:12 conditions with the light on from 8:00 a.m. (this timing set as zeitgeber time 0 [ZT0]) to 8:00 p.m. (ZT12). Mice in poor health were excluded from the study. In vivo experiment, 10 μl of rAAV (1×10^{11} GC; 5×10^{12} GC/kg) was injected into mouse knee joints. All rAAV was purchased from OBiO Tech (Shanghai), GENECHEM (Shanghai), and HuaMeng (Wuhan). IN-1130 (MCE, HY-18758, 10 mg/kg, daily) was injected intraperitoneally into the mice. NRS (MCE, HY-N2253, 3 mg/kg/d) was intragastrically administered to the mice. Recombinant ADAMTS4 (rADAMTS4, Merck Millipore, CC1028, 4 μl volume containing 10 ng rADAMTS4 per knee joint) was injected into mouse knee joints. ADAMTS4 inhibitor (MCE, HY-114996, 4μl volume containing 10μM rADAMTS4 per knee joint) was injected into mouse knee joints to inhibit ADAMTS4 activity in vivo, according to previous study(1, 2). Our study examined male and female animals, and similar findings are reported for both sexes. All animal studies were performed on mice and approved by Huazhong University of Science and Technology Laboratory Animal Centre and Use Committee (IACUC number: 3293).

Flow Cytometry

For bone marrow flow cytometric analysis, we collected femurs and tibiae after euthanizing mice, removed epiphysis, muscles, and periosteum around the bone, and then crushed the metaphysis and diaphysis regions of the bone in ice-cold PBS to get the bone marrow. Then we used Collagenase Type I (3 mg/mL; Biosharp, BS163-1g) and Dispase (4 mg/mL; Roche Diagnostics, Cat#4942078001) to digest whole bone marrow at 37 ℃ for 30 min to obtain single-cell suspensions. After filtration and washing, the cells were counted and incubated for 45 min at 4 ℃ with PE-conjugated anti-CD31 antibody (Biolegend, 102408), FITC-conjugated anti-CD45 antibodies (BD, 553079), APC-Cy™7-conjugated anti-CD45 antibodies (BD, 557659), APC-conjugated anti-Ter119 antibodies (Proteintech, APC-65149), BV605-conjugated anti-Sca-1 antibodies (Biolegend, 108133), PerCP-Cy5.5-conjugated anti-CD11b antibody (Biolegend, 101230), PE-conjugated anti-CD29 antibody (ThermoFisher, 12-0291-81), FITC-conjugated anti-CD3 (Biolegend, 102407), or BV510-conjugated Zombie Aqua™ (Biolegend, 423101). Then we used a Fixation/Permeablization Kit (BD, 554714) for cell fixation and permeabilization. After washing, the cells were incubated with BV405-conjugated anti-BMAL1 antibodies (NOVUS BIOLOGICALS, NB100-2288AF405). The cells were then analyzed by a BD Flow Cytometer (BD LSRFortessa™X-20 Special Order Product).

Isolation and Culture of Mouse Bone Marrow Endothelial Cells and Bone Marrow Mesenchymal Stem Cells

The ECs used in this study were mouse bone marrow ECs from *Bmal1*$^{fl/fl}$ mice injected with rAAV-*ctrl* or rAAV-*Cdh5*-Cre. As described above, we used Collagenase Type I and Dispase to digest whole bone marrow to obtain single-cell suspensions. After filtration and washing, the cells were incubated with CD31 MicroBeads (Miltenyi Biotec, 130-097-418) and CD45 MicroBeads (Miltenyi Biotec, 130-052-301) respectively, and CD31+ CD45- cells were selected, according to the operating instructions of MicroBeads. Sorted cells were cultured in endothelial cell growth medium (EBM-2, Clonetics; Lonza) supplemented with EGM-2 Single Quots (CC-4176, Clonetics; Lonza) and the adherent cells were determined as ECs. Cells were fed every third day. Cultures were maintained at 37°C with 5% CO_2 in a humidified atmosphere. The ECs were used directly for in vitro experiments and subsequent qPCR, Western blot, or immunofluorescence staining.

For mouse BMSC isolation, we digested bone marrow to obtain single-cell suspension from the tibia and femur of 3-week-old mice. The cells were sorted through density gradient centrifugation, according to the operating instructions of animal BMSC isolation (Solarbio, P8600). Then the cells were incubated with CD31 MicroBeads (Miltenyi Biotec, 130-097-418), and CD31- cells were selected as BMSCs. We cultured BMSCs in αMDM containing 10% fetal calf serum (Thermo Fisher Scientific) at 37 °C in a humidified atmosphere with 5% CO_2. The medium was changed every 3 days. BMSCs were used directly for in vitro experiments and subsequent colony formation assays, CCK8 assays, EdU staining, adipogenic and osteogenic differentiation assays, flow cytometry for cell cycle, ROS levels, and apoptosis analysis.

RNA-seq and analysis

Bone marrow cells extracted from femurs and tibias of mice injected rAAV-*ctrl* or rAAV-*shBmal1* were labeled with PE-conjugated anti-CD31 antibody (Biolegend, 102408), FITC-conjugated anti-CD45 antibodies (BD, 553079), and APC-conjugated anti-Ter119 antibodies (Proteintech, APC-65149). Then BMCEs were isolated from bone marrow cells by flow cytometry. Total RNA was extracted from BMCEs isolated from mice injected rAAV-*ctrl* or rAAV-*shBmal1* using Trizol reagent according to the manufacturer's protocol. The samples were sent to Labs SEQHEALTH (Wuhan, China) for quantification, preparation of the RNA-seq library, and sequencing. Sequencing was performed on an Illumina Hiseq X Ten second-generation sequencing platform (Supplementary Table 3). Genes with adjusted P-value <0.05 and Fold-change ≥ 1.5 were assigned as differentially expressed. Gene ontology (GO) enrichment analysis and Kyoto Encyclopedia of Genes and Genomes (KEGG) enrichment analysis were performed using R package clusterProfiler(3) (Supplementary Table 4). Gene expression was quantified using RPKM values. RNA-seq data have been uploaded in National Center for Biotechnology Information’s Sequence Read Archive (SRA) database (BioProject number: PRJNA1165254).

Micro-CT

Femurs were harvested and fixed from the mice without soft tissues. Then the bones were scanned at a resolution of 9 μm using the Micro-CT (SkyScan 1176, Broker). The images were used to reconstruct and quantify by InstaRecon/NRecon Research

Workplace software. Bone volume/total volume, trabecular thickness, bone surface area/bone volume, trabecular number, and trabecular separation of femurs were indexes to show differences among mice.

Telomere-Associated Foci (TAF)

To measure osteocyte senescence, as published in literature(4), the TAF assay was performed on non-decalcified methacrylate-embedded sections of femurs isolated from C57BL/6 mice with indicated treatment. Bone sections were deplasticized and hydrated with gradients of ethanol followed by washes in H_2O and PBS. Antigen retrieval was achieved by Tris-EDTA incubation (pH 9.0) at 95 °C for 15 min. Following cool-down and then hydration with H_2O and PBS (0.5% Tween-20/0.1% Triton X-100), slides were placed in a blocking buffer at RT for 1 h. After dilution in the blocking buffer, the primary anti-γ-H2AX antibody (1:200; Abclonal, AP0687) was incubated at 4 °C overnight. Next morning, after washing, slides were incubated with fluorescent secondary antibody (1:200) in the blocking buffer at 4 °C for 1 h. Subsequently, slides were washed (three times) with PBS, followed by FISH for the detection of TAF. Briefly, following 4% paraformaldehyde cross-linking for 20 min, sections were washed with PBS and dehydrated in graded (70%, 90%, 100%) ice-cold ethanol (three min each). Sections were then dried/denatured for 10 min at 80 °C with hybridization buffer that contained 0.1 M Tris (pH 7.2), 25 mM MgCl2, 70% formamide (Millipore Sigma), 5% blocking reagent (Roche), and 1.0 μg/mL of TAMRA-labeled telomere-specific (CCCTAA) peptide nucleic acid probe (Sangon Biotech). This was followed by hybridization in a humidified dark room at RT for 2 h. Sections were stained with DAPI

for 10 min at room temperature. Finally, the images were observed with a laser scanning confocal microscope (Nikon, Japan) and NIS-Elements Viewer software. TAF number per osteocyte was quantified by examining the overlap (yellow) of signals from the telomere probe (red) with the γ-H2AX (green) *i.e.* phosphorylated C-terminal end of histone H2A.X, thus marking double-strand DNA breaks. The average number of TAF per osteocyte (OCY) was quantified using ImageJ, whereas the percentage (%) of TAF+ OCYs for each mouse was calculated using the following criteria: % of cells with ≥1 TAF, % of cells with ≥2 TAF, and % of cells with ≥3 TAF.

Histology and Immunohistochemistry Staining

Fresh femurs were dissected from mice, 4% paraformaldehyde, and decalcified by 20% EDTA solution. The decalcified specimens were prepared for a four-micrometer-thick paraffin section or twenty-micrometer-thick frozen section(5, 6). TRAP staining and H&E staining were performed on the standard protocols. Immunofluorescence was performed using DYKDDDDK tag antibody (Proteintech, 66008-4-Ig, 1:2000), anti-Cre Recombinase antibody (CST, 15036, 1:800), 488-conjugated anti-CD31 antibody (R&D, FAB3628G-100, 1:50), anti-BMAL1 antibody (Invitrogen, PA1-523, 1:200), anti-RUNX2 antibody (Abcam, ab192256, 1:200), anti-OSX antibody (Abcam, ab209484, 1:200), anti-ADAMTS4 antibody (Abcam, ab185722, 1:200), anti-MMP2 antibody (Proteintech, 10373-2-AP, 1:200), anti-MMP9 antibody (Proteintech, 10375-2-AP, 1:200), anti-FBN1 antibody (ABclonal, A16677, 1:200), anti-SMAD3 antibody (ABclonal, A19115, 1:200), and/or fluorescent secondary antibody (1:200) was then added for 1 h. Digital images were taken with a laser scanning confocal microscope

(Nikon-si-A1, Japan) and NIS-Elements Viewer software (VERSION 5.21.00).

To examine the leakage of blood vessels, we anesthetized the mice and injected Dextran-FITC (70 kDa, ThermoFisher) through tail vein. After 10 min, the mice were injected into the heart with rehydration salts containing heparin, and then the femur specimens were taken, decalcified at 4℃ under dark condition, and made into frozen sections. Frozen sections were incubated with anti-CD31 antibody (Abcam, ab7388) and fluorescent secondary antibodies, as well as DAPI, to evaluate vascular permeability by confocal microscopy.

Thsd4 or *Adamts4* FISH probe synthesized by Ribo Bio Tech (Guangzhou, China). FISH was performed on the FISH kit protocol (Ribo Bio Tech). Frozen mouse femur sections were incubated with a pre-hybridization buffer at 37 ℃ for 30 min. Subsequently, frozen sections were hybridized with 20 μM using Cy3-labeled RNA of *Thsd4* or *Adamts4* FISH probe mix in a moist chamber at 37 ℃ overnight. Frozen sections were washed thrice in 4 × SSC with 0.1% Tween-20 for 5 min at 42 ℃, followed by rinsing once for 5 min at 42 ℃ in 2 × SSC and then rinsing once for 5 min at 42 ℃ in 1 × SSC. After hybridization, frozen sections were stained with DAPI for 10 min at room temperature. Finally, the images were observed with a laser scanning confocal microscope (Nikon, Japan) and NIS-Elements Viewer software.

Viral Infection and siRNA Transfection

For knockdown *Bmal1*, we performed the protocol as our previous study(7). For the knockdown of *Adamts4*, *Col5a2*, *Csf3*, *Il34*, and *Tfpi2*, siRNAs were utilized according

to the manufacturer's protocols. For overexpression, primary mouse bone marrow ECs were transfected with Lentivirus gene expression vector (3rd generation) containing *Arntl* (NM_001243048.1), *Thsd4* (NM_172444.4) or *Adamts4* (NM_172845.3) gene sequence listed in Supplementary Table 2.

Co-culture experiments of ECs and BMSCs

As for indirect co-culture, ECs ($6x10^4$ cells) were seeded on the upper chamber (3 μm pore; Corning Life Sciences) and infected with siRNAs (Supplementary Table 1) or lentivirus (Supplementary Table 2), according to the experimental requirements. Then the upper chamber containing ECs was moved to the bottom chamber, which was seeded BMSCs ($6x10^4$ cells) within 250μl Matrigel (BD, 354234). Every 7 days, ECs in the upper chamber were replaced with new ECs infected with siRNAs. BMSCs in the bottom chamber were used to perform senescence-associated SA-β-gal (5 days co-culture) staining and EdU staining (Beyotime, C0071S) (2 days co-culture) according to the standard protocols. After four weeks of co-culture in the osteogenic induction medium, BMSCs in the bottom chamber were used for ARS staining performed on the standard protocols. After three weeks of co-culture in the lipogenic induction medium, BMSCs in the bottom chamber were used for Oil Red O staining using standard protocols. For colony formation assay, 500 BMSCs were seeded on the bottom chamber and co-cultured with the ECs for 3 weeks. Then BMSCs were stained with crystal violet. For CCK8 assays, ECs ($6x10^4$ cells) were seeded on the upper chamber, while BMSCs ($3x10^4$ cells) were seeded on the bottom chamber. After co-culturing for 48, 72, and 96 h, the upper chamber was removed and CCK8 detection was performed on the BMSCs

in the lower chamber according to the instructions.

For cell cycle assays, ROS assays, and cell apoptosis assays, ECs ($6x10^4$ cells) were seeded on the upper chamber, while BMSCs ($3x10^4$ cells) were seeded on the bottom chamber. After 2 days of co-culturing, BMSCs were digested and collected for cell cycle assays (Beyotime, C1052), ROS assays (Beyotime, S0033S), and cell apoptosis assays (Abclonal, RK05875) according to the kit manuals.

To detect changes in ADAMTS4 in ECs, ECs ($1x10^4$ cells) were seeded on the bottom chamber, while BMSCs were seeded on the upper chamber within Matrigel. After two days of co-culture, ECs in the bottom chamber were used to perform immunofluorescence with anti-ADAMTS4 antibody (Abcam, ab185722, 1:200) or qRT-PCR.

The reagents used in cell experiments include GW4869 (MCE, HY-19363, 5 μM), imipramine (MCE, HY-B1490A, 5 μM), rTHSD4 (recombination THSD4, AtaGenix, Wuhan, 2.5 μg/ml), rADAMTS4 (R&D, 4307-AD-020, 200 ng/ml), TGFβ neutralizing antibody (ThermoFisher, 16-9243-85, 20 μg/ml), (E)-SIS3 (MCE, HY-13012, 5 μM), RepSox (MCE, HY-13013, 10 μM), and Caerulomycin A (CaeA, MCE, HY-114495, 0.15 or 0.3 μM). rTHSD4 was produced by mammalian cells expression system and the target *Thsd4* gene encoding Gln27-Arg1018 was expressed with C-HisTag.

Quantitative Reverse Transcription (qRT)-PCR Analysis

Total RNA was extracted via Trizol (Takara, Tokyo, Japan) according to the manufacturer's protocols. cDNA synthesis was done with oligo dT primers and reverse

transcriptase (Takara). Real-time RT–PCR was performed using SYBR Green PCR protocol and the BIO-RAD CFX Connect^{TM} Real-Time system (Bio-Rad Laboratories Inc, USA). Relative mRNA expression was normalized by GAPDH using the 2-△△Ct method. Primers used for amplification are listed in Supplementary Table 1.

Single-Cell Sequencing Analysis

Single-cell sequencing data of the adult human bone marrow were obtained from the Gene Expression Omnibus (GSE169396) database. Seurat R package (R-4.3.2, Seurat 5.0.1) was then used to control the quality of each cell (nFeature_RNA > 200 & nFeature_RNA < 5000 & percentage mt <15). After processing the data using NormalizeData, the FindVariableFeature function was applied to screen feature genes for subsequent Principal Component Analysis (PCA). The first ten PCAs were selected to determine the clusters. Dimensionality reduction analysis and cluster visualization were performed using the uniform manifold approximation and projection method. The FindAllMarkers function was used to identify markers in the clusters. GSM5201885 sample was removed due to undetectable level for *Bmal1*, and the Merge function and harmony function were applied to the remaining three samples. After naming each cluster based on markers, the mean expression of *Bmal1* was calculated for each cluster.

Immunosorbent Electron Microscope (ISEM)

Bone marrow tissue was collected from freshly culled femurs and placed into sterile PBS. For ultrastructural observation, the tissue was fixed at room temperature for 2 h in 2.5% glutaraldehyde in cacodylate buffer. For transmission electron microscopy,

samples were washed with cacodylate buffer and post-fixed for 1 h at room temperature in 1% osmium tetroxide in cacodylate buffer, dehydrated in a graded series of ethanol, and then embedded in Epon 812 (SPI Supplies) using acetone as an intermediate solvent. Specimens were sectioned into 50–70 nm thick ultrathin sections on an LKB ultramicrotome with a diamond knife. Immunolabelling was performed with an anti-rabbit FBN1 antibody (Abclonal, A16677, titer 1:10), labeled with colloidal gold. Immunogold-sliver staining was carried out with anti-rabbit TGFβ antibody (Bioss, bs-4538R, titer 1:10) and anti-mouse TGFβRII antibody (Proteintech, 66636-1-Ig, titer 1:10), labeled with colloidal gold and silver. Analysis was carried out using a JEOL JEM 1230 transmission electron microscope.

Cleavage of FBN1 by ADAMTS4 in vitro

0.2 μg rADAMTS4 (R&D, 4307-AD-020) was incubated with 1.0 μg recombinant fibrillin-1 fragments (R&D, 10224-FI-050) in 50 mM Tris-HCl pH 7.5. Following incubation for 24 h and 48 h at 37 °C, the samples were subjected to analysis by SDS-PAGE using a polyacrylamide concentration of 15% and silver staining.

ChIP-Seq and ChIP Assay

Chromatin immunoprecipitation was performed per the manufacturer's protocol (Beyotime, ChIP Assay Kit, P2078). Briefly, 1×10^6 cells were fixed with 37% formaldehyde for 10 min, glycine was then added to a final concentration of 0.125 mol/L to quench the reaction. Cells were washed twice with ice-cold phosphate-buffered saline, lysed, and then sonicated to 200-400 bp of DNA fragments. The

chromatin was then incubated overnight with antibody against to BMAL1 (Abcam, ab3350) or SMAD3 (Abcam, ab202445). Precipitated ChIP DNA and input DNA were washed, reverse-crosslinked, and digested with proteinase K and RNase A. The DNA samples were then purified with spin columns and sent for sequencing (Wuhan Ruixing Biotechnology). Some DNA samples were reserved for qPCR with primers (Supplementary Table 1). ChIP-seq data have been uploaded in National Center for Biotechnology Information's Sequence Read Archive (SRA) database (BioProject number: PRJNA1165972).

Luciferase Reporter Assay

The following luciferase assay experiments were performed on primary ECs (1×10^5 cells/well in 24-well plates). 375ng firefly luciferase reporter vectors and 125 ng Renilla luciferase reporter vectors pGL-3.0 basic were co-transfected into cells using Lipo6000 (Beyotime) as instructed. After 48-hours cells were extracted. Firefly and Renilla luciferase activities were consecutively measured using a dual luciferase reporter assay system (Promega). The luciferase signals were standardized according to the firefly/Renilla ratio to confirm the transcriptional activities of *Thsd4* and *Adamts4* promoters.

Three-point Bending Test.

To measure the cortical strength of the femurs, the mechanical-testing machine equipped with a Zwick Z020 (ZwickRoell) was used to run a 3-point bending test. There are two end support points and one central loading point for the 3-point bending test.

The femurs were placed on two supports separated by a distance of 9 mm, and the load was applied to the midpoint of the shaft. Each bone was loaded at a constant speed of 0.05 mm/s^{-1} until failure. The biomechanical measurement data were collected from the load-deformation curves. The maximum load (N) and stiffness (N/mm) were recorded.

Immunoblotting

Protein was extracted with RIPA lysis buffer containing protease and phosphatase inhibitors (Beyotime Biotechnology, Wuhan, China). An equal amount of protein from each specimen was subjected to electrophoresis on 4% or 10% sodium dodecyl sulfate-polyacrylamide gel and subsequently transferred onto the PVDF membrane. The membranes were incubated overnight with primary antibodies, including anti-BMAL1 antibody (Abcam, ab93806, 1:2000), anti-RUNX2 antibody (Abcam, ab192256, 1:1000), anti-OSX antibody (Abcam, ab209484, 1:1000), anti-P21 antibody (Abclonal, A19094, 1:1000), anti-THSD4 antibody (Abclonal, A17773, 1:1000), anti-ADAMTS4 antibody (Abcam, ab185722, 1:1000), anti-SMAD3 antibody (Abcam, ab202445, 1:1000), anti-phospho-SMAD3 antibody (Abclonal, AP0727, 1:1000), anti-FBN1 antibody (Abclonal, A16677, 1:1000), or anti-GAPDH antibody (Proteintech, 10494-1-AP, 1:10000) at 4 °C. The membranes were next incubated with biotinylated secondary antibodies (Proteintech), followed by exposing in the appearance of the Western Blotting Detection Kit (GE Healthcare, cat#: RPN2106). The protein statistics of BMAL1 were based on three bands.

Statistical Analysis

All data are displayed as means ± SD. GraphPad Prism v. 9.0.2 was used for the statistical analyses. Data of Figure 4G, 6D and 6I were analyzed using DiscoRhythm (https://mcarlucci.shinyapps.io/discorhythm/). Each curve refers to normalized mean densitometry values of a proteasome protein from the western blots. The curves refer to the average trend of diurnal changes in p-SMAD3 or P21 protein levels, indicating the best fit to the points by cosinor analysis. P-values indicate regression analysis significance. The other data were evaluated by a unpaired t-test with Welch's correction, by One-way ANOVA, Tukey's multiple comparisons tests, or by two-way ANOVA, multiple comparisons. A p-value <0.05 was considered significant. All source numerical data were listed in statistical source data.

References

1. Chen P, Zhu S, Wang Y, Mu Q, Wu Y, Xia Q, et al. The amelioration of cartilage degeneration by ADAMTS-5 inhibitor delivered in a hyaluronic acid hydrogel. *Biomaterials.* 2014;35(9):2827-36.
2. Gilbert AM, Bursavich MG, Lombardi S, Georgiadis KE, Reifenberg E, Flannery CR, et al. 5-((1H-pyrazol-4-yl)methylene)-2-thioxothiazolidin-4-one inhibitors of ADAMTS-5. *Bioorg Med Chem Lett.* 2007;17(5):1189-92.
3. Yu G, Wang L-G, Han Y, and He Q-Y. clusterProfiler: an R package for comparing biological themes among gene clusters. *Omics : a Journal of Integrative Biology.* 2012;16(5):284-7.
4. Farr JN, Saul D, Doolittle ML, Kaur J, Rowsey JL, Vos SJ, et al. Local senolysis in aged mice only partially replicates the benefits of systemic senolysis. *J Clin Invest.* 2023;133(8).
5. Kusumbe AP, Ramasamy SK, Starsichova A, and Adams RH. Sample preparation for high-resolution 3D confocal imaging of mouse skeletal tissue. *Nat Protoc.* 2015;10(12):1904-14.
6. Yang M, Li CJ, Sun X, Guo Q, Xiao Y, Su T, et al. MiR-497 approximately 195 cluster regulates angiogenesis during coupling with osteogenesis by maintaining endothelial Notch and HIF-1alpha activity. *Nat Commun.* 2017;8:16003.
7. Zhao J, Zhou X, Tang Q, Yu R, Yu S, Long Y, et al. BMAL1 Deficiency Contributes to Mandibular Dysplasia by Upregulating MMP3. *Stem Cell Reports.* 2018;10(1):180-95.
