# Supplementary material for: Endothelial BMAL1 decline during aging leads to bone loss by destabilizing extracellular fibrillin-1
Source: J Clin Invest. 2024 Dec 16;134(24):e176660. doi: 10.1172/JCI176660 (PMC11645155; doi:10.1172/JCI176660)

Full unedited blots for Figure 1A

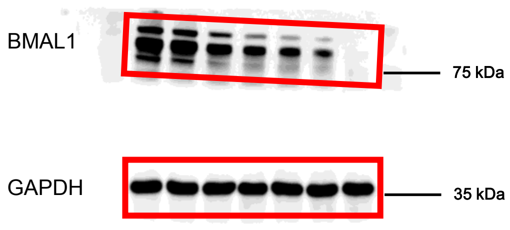

Full unedited blots for Figure 1I

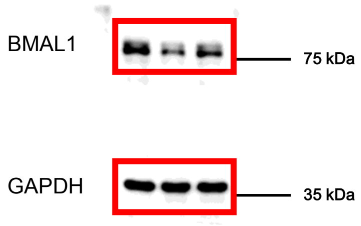

Full unedited blots for Figure 1D

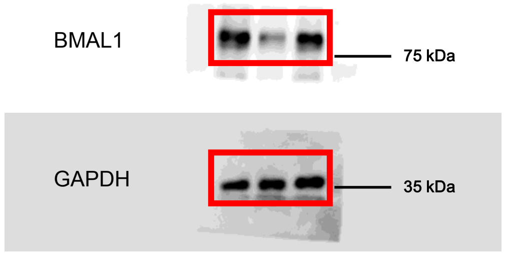

Full unedited blots for Figure 3C

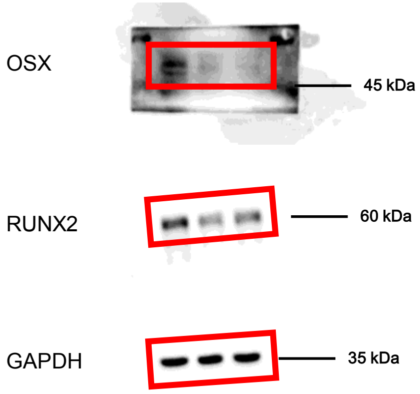

Full unedited blots for Figure 3F

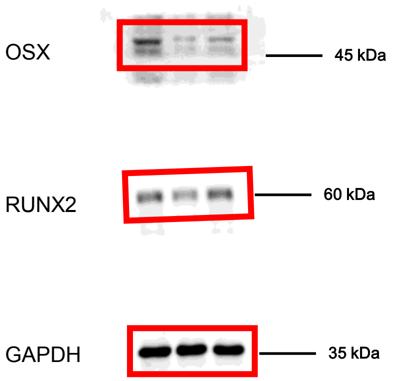

Full unedited blots for Figure 4G

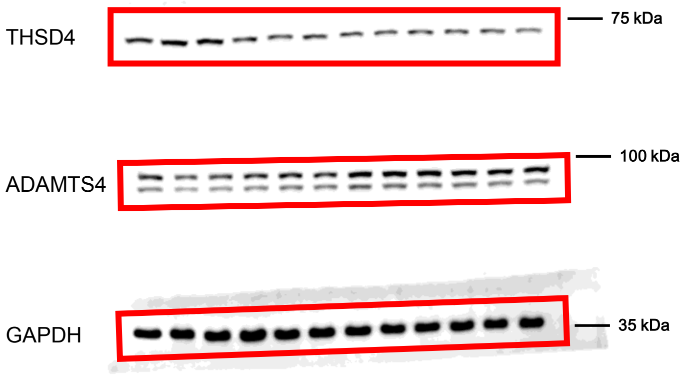

Full unedited blots for Figure 4H

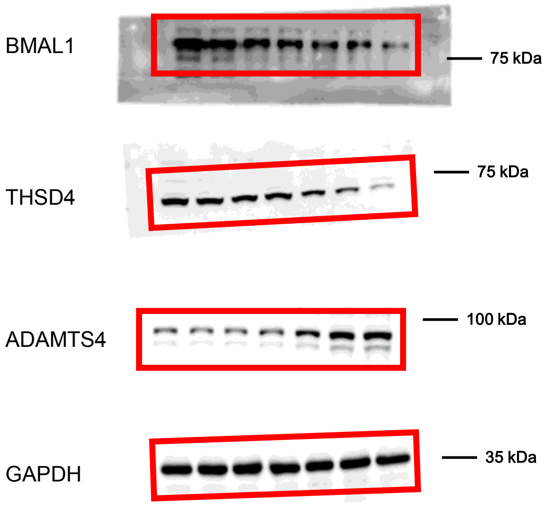

Full unedited blots for Figure S6B

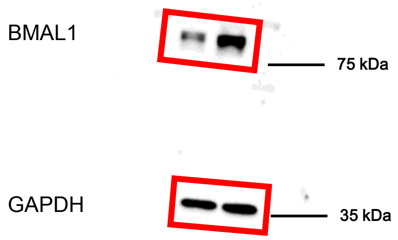

Full unedited blots for Figure S7F

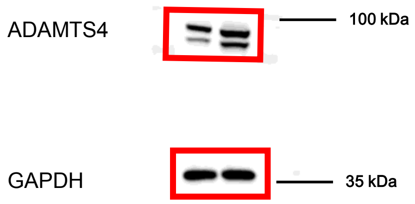

Full unedited blots for Figure S7B

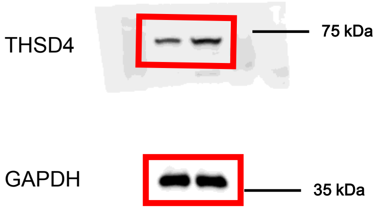

Full unedited blots for Figure S9G

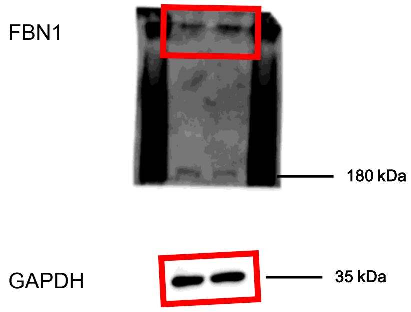

Full unedited blots for Figure 6D

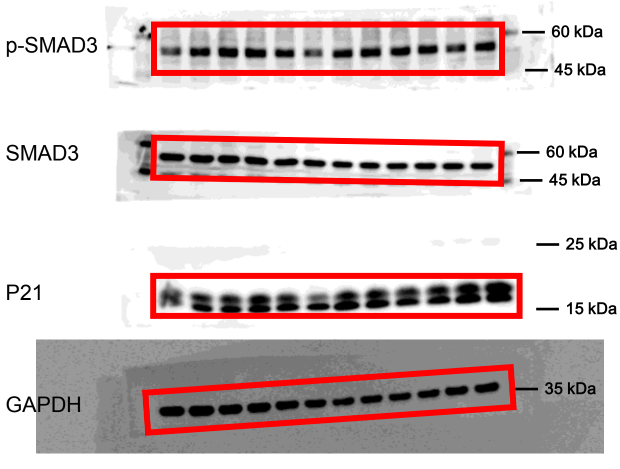

Full unedited blots for Figure 6E

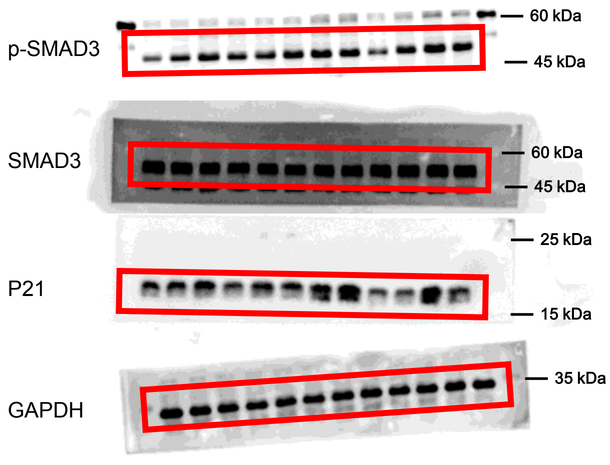

Full unedited blots for Figure 6I

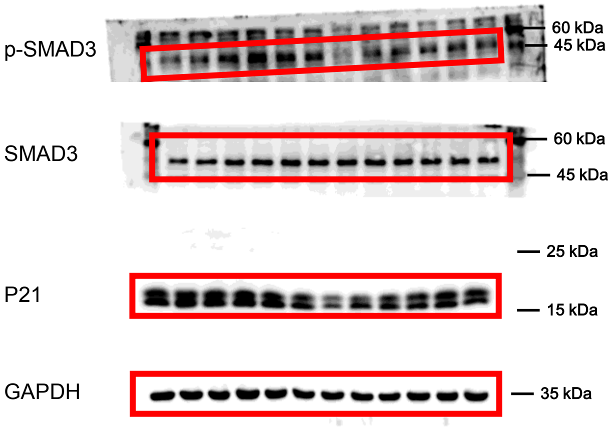

Full unedited blots for Figure 7B

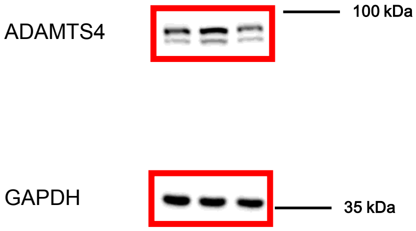

Full unedited blots for Figure 7I

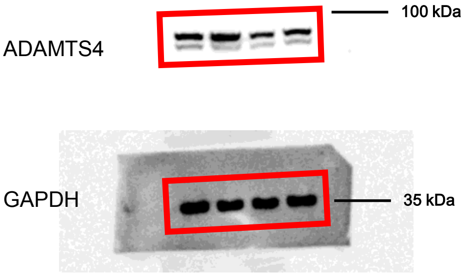

Full unedited blots for Figure 7H

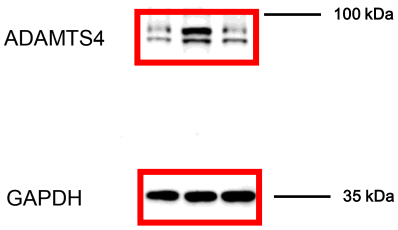

Full unedited blots for Figure S11D

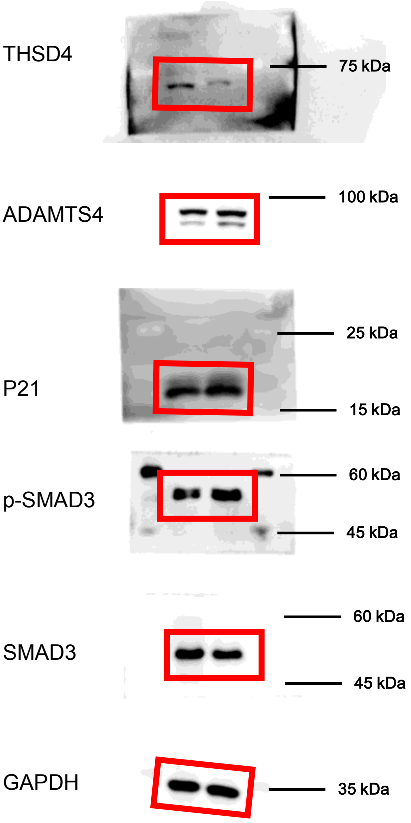

Full unedited blots for Figure 7J

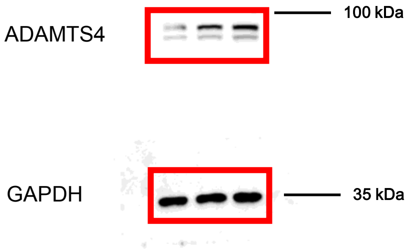

Full unedited gels for Figure S8C

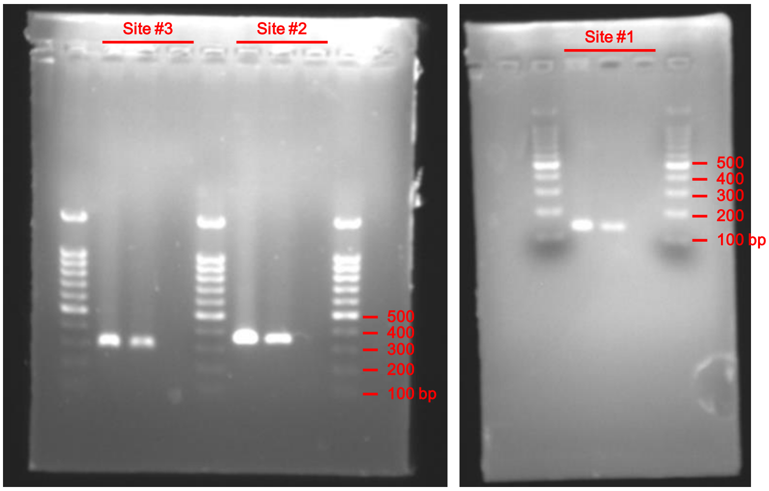

Full unedited gel for Figure 7M

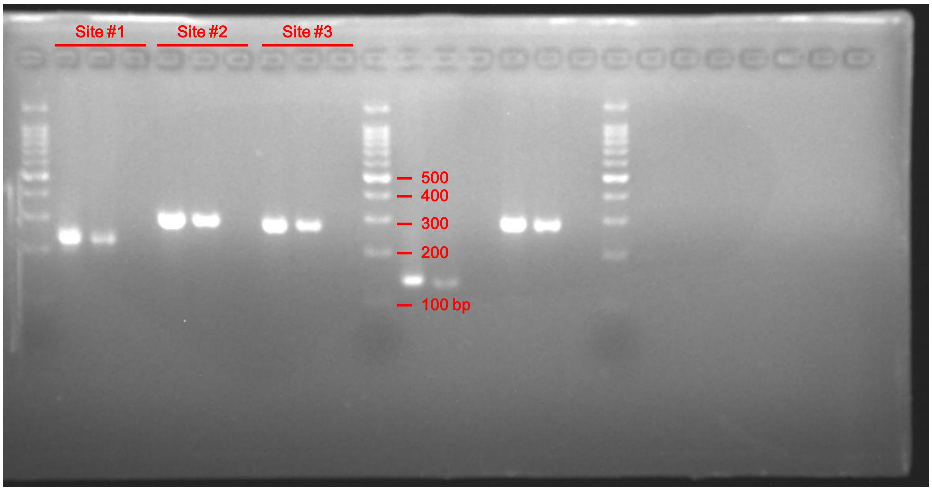

Full unedited gel for Figure S9B

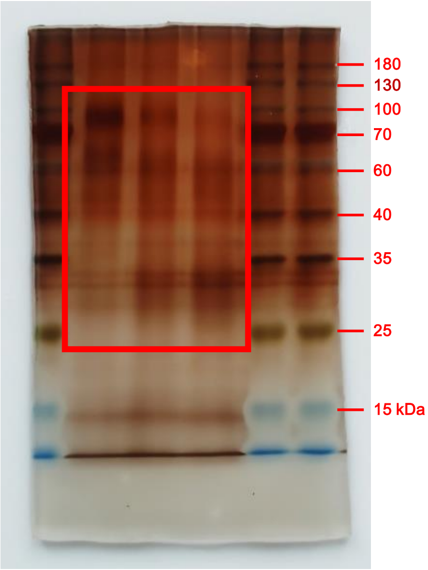

Supplement: Unedited blot and gel images [file jci-134-176660-s073.pdf]
